# Matching the right study design to decision-maker questions: Results from a Delphi study

**DOI:** 10.1371/journal.pgph.0002752

**Published:** 2024-02-29

**Authors:** Cristián Mansilla, Gordon Guyatt, Arthur Sweetman, John N Lavis

**Affiliations:** 1 McMaster Health Forum, McMaster University, Hamilton, Ontario, Canada; 2 Health Policy PhD program, McMaster University, Hamilton, Ontario, Canada; 3 Department of Health Research Methods Evidence and Impact, McMaster University, Hamilton, Ontario, Canada; 4 Department of Economics, Faculty of Social Sciences, McMaster University, Hamilton, Ontario, Canada; University of Ghana College of Health Sciences, GHANA

## Abstract

Research evidence can play an important role in each stage of decision-making, evidence-support systems play a key role in aligning the demand for and supply of evidence. This paper provides guidance on what type of study designs most suitably address questions asked by decision-makers. This study used a two-round online Delphi approach, including methodological experts in different areas, disciplines, and geographic locations. Participants prioritized study designs for each of 40 different types of question, with a Kendall’s W greater than 0.6 and reaching statistical significance (p<0.05) considered as a consensus. For each type of question, we sorted the final rankings based on their median ranks and interquartile ranges, and listed the four study designs with the highest median ranks. Participants provided 29 answers in the two rounds of the Delphi, and reached a consensus for 28 (out of the 40) questions (eight in the first round and 20 in the second). Participants achieved a consensus for 8 of 15 questions in stage I (clarifying a societal problem, its causes, and potential impacts), 12 of 13 in stage II (finding options to address a problem) and four of six in each of stages III (implementing or scaling-up an option) and IV (monitoring implementation and evaluating impact). This paper provides guidance on what study designs are more suitable to give insights on 28 different types of questions. Decision-makers, evidence intermediaries (, researchers and funders can use this guidance to make better decisions on what type of study design to commission, use or fund when answering specific needs.

## Introduction

There has been a growing recognition of the potential for evidence to support many aspects of decision-making processes. Evidence can play a significant role in clarifying problems and their causes, analyzing potential solutions, supporting implementation, and monitoring implementation, and evaluating impacts [1–3].

During the COVID-19 pandemic, evidence provided multiple insights critical to questions asked by decision-makers [4]. The lessons learned from this global crisis led several organizations to call for formalizing and strengthening evidence-support systems and the global evidence architecture [5–7].

Robust evidence-support mechanisms require alignment between decision-makers’ needs (evidence demand) and the work of evidence producers (evidence supply) and intermediaries (i.e., people working in between researchers and decision-makers) (evidence supply). Typically, a given need can best be met with a combination of different forms of evidence. When answering complex questions, decision-makers often require a combination of different forms or lines of evidence [8]. For example, a policymaker may use quantitative data from a randomized controlled trial to describe the likely benefits and harms of implementing a particular policy intervention, and may also rely on qualitative data to gain a deeper understanding of how the public might perceive the intervention.

Following the policy cycle used in Update 2023 from the Global Commission on Evidence to Address Societal Challenges, previous research efforts generated a taxonomy of decision-makers’ questions grouped into four decision-making stages (S1 Fig). This taxonomy can help decision-makers to frame their need for evidence in a form that an evidence intermediary or producer can answer with different forms of evidence.

After identifying a clear decision-making need in the form of a question, evidence intermediaries and producers can tailor their evidence support to address specific issues and share evidence that is more likely to be used in decision-making processes. The current study builds on the previously created taxonomy of decision-maker questions and matches questions to study designs. While some have suggested a single evidence hierarchy with randomized trials and evidence syntheses at the top of an evidence pyramid [9], our approach aligns with work that presents a more sophisticated approach that recognizes that the preferred study design will differ based on the specific type of question being asked [10, 11].

This paper provides guidance on what type of study designs can address each decision-maker question. Specifically, it:

1. lists specific study designs that would provide some insights to address different types of question;
2. provides a ranking of study designs that would be most suitable to answer these questions.

## Materials and methods

This is a Delphi study of methodological experts in different areas and disciplines.

### Participants

Methodological experts—from the six WHO regions (Africa, Americas, Eastern Mediterranean, Europe, South East Asia, and the Western Pacific) representing the eight different forms of evidence that were identified by the Global Commission on Evidence to Address Societal Challenges (data analytics, modelling, evaluation, behavioural/implementation research, qualitative insights, evidence syntheses, technology assessment/cost-effectiveness analysis and guidelines, and experience in the health sector and in other sectors) were identified through global networks (e.g., EVIPNet, Cochrane and GIN). The study team obtained contact information through publicly available sources, and purposively sampled experts using the above criteria. Between 11 April and 16 May 2023, they were invited to participate in this online Delphi study.

### Data collection

We used an online questionnaire to ask participants to rank the suitability of study designs to answer different types of question. The questionnaire provided 40 different types of questions (S1 Fig provides the entire taxonomy of types of question used in this study).

In both Delphi rounds, participants considered a list of study designs potentially relevant to each type of question, and prioritized the designs’ suitability to answer the question. To facilitate understanding, a brief explanation of each type of question and some examples were also provided. A glossary of study designs was also made available for participants, and they could declare that they did not have enough expertise to answer a given question.

In the first round, participants prioritized at least four study designs and suggested any additional study designs they considered to be missing from the original list. They could choose to work through the complete list of types of question or, depending on their expertise in the eight forms of evidence mentioned above, work through a subset of questions.

Using a ranking-type Delphi process [12, 13], we used the level of consensus reached in round one to prioritize the questions included in the round two. Questions that reached a consensus in the first round were not included in the second round. In the second round, participants either confirmed the previous rank order from round 1 or suggested an alternative ranking. All participants in the second round answered the complete list of question types.

### Statistical analysis

Responses were analyzed in each round using median ranks and distributions (interquartile ranges). Additionally, in each round, Kendall’s W (a statistical measure of consensus in ranking-type surveys) was used to measure consensus [12–15]. Answers were considered if the participants ranked at least two options and did not declare that they had insufficient expertise.

The ranking given by each participant was classified ordinally (e.g., first priority received a 1, second priority received a 2, etc.) and, since not every participant ranked all the options (they were asked to prioritize at least four options), non-ranked values were imputed as the average value between the latest item ranked and the greatest possible rank for each answer (e.g., if one participant ranked four of the six available options, the two options that were not ranked both received a value of 5.5). From these rankings, medians and interquartile ranges were calculated for each study design, and the Kendall’s W with ties (considering missing values as ties, as suggested by Kendall & Gibbons [14]) was calculated, along with their statistical significance, using the Real Statistics Resource Pack for Microsoft Excel [15].

The study designs were sorted for each type of question based on their median ranks (from smallest to largest). If more than one option had the same median rank, then the smallest number of the 25^th^ percentile, followed by the smallest number of the 75^th^ percentile, was used to sort these options.

One question was considered to have reached consensus when Kendall’s W was greater than 0.6 and statistically significant (p-value < 0.05). The options ranked lower in the list with its IQR suggesting no change in their ranking position (e.g., in a question of 7 study design options, a cross-sectional study had a median rank of 7, and a IQR from 7 to 7) were not included in the second round. Suggestions of additional study designs collected from the first round were, when appropriate, included in the study designs to be ranked in the second round.

In each question, a study design whose median rank was 1, and its interquartile range does not alter its ranking as first were identified and placed as gold standard. Similarly, a study design whose median rank was last, and its interquartile range does not alter its ranking as last was also identified.

### Ethics statement

The Hamilton Integrated Research Ethics Board (HiREB), Project ID: 14691 approved the study. Participants received an information sheet including the ethical considerations of being part of this study, and their response was considered as implicit consent, as their responses were treated anonymously.

## Results

Seventy-three methodological experts were initially identified as potentially eligible to participate and, to balance the sampling across WHO regions and forms of evidence, an invitation was sent to 46 individuals in the first round. Two participants declined the invitation to participate in the study, and were replaced with two from the original list. Twenty-one participated in round 1 (response rate 46%), while 8 participated in round 2 (response rate 18%). In total, 29 answers were received in both rounds of the Delphi process.

Table 1 presents the participants’ characteristics, including their WHO region and the forms of evidence assigned to them for each Delphi round. In the first round, most of the participants were from the Americas and Europe, while no experts participated from South-East Asia, and only one from the Eastern Mediterranean. In the second round, most of the participants were based in the Americas, while no participants were from South-East Asia or the Eastern Mediterranean.

**Table 1:** Characteristics of the participants included in the study per each round (n = 21 for round 1; n = 8 for round 2), ranked by frequency in round 1.

	Round 1		Round 2	
	N	%	N	%
**WHO region**				
Americas	8	38%	4	50%
Europe	5	24%	1	13%
Africa	4	19%	1	13%
Western Pacific	3	14%	2	25%
Eastern Mediterranean	1	5%	0	0%
South-East Asia	0	0%	0	0%
**Form of evidence of expertise** *				
Evidence syntheses	17	81%	7	88%
Behavioral/implementation research	13	62%	4	50%
Guidelines	12	57%	5	63%
Evaluation	11	52%	1	13%
Data analytics	7	33%	1	13%
Modelling	6	29%	1	13%
Qualitative insights	6	29%	3	38%
Technology assessments/cost-effectiveness analyses	4	19%	1	13%

*Participants could have expertise in more than one form of evidence. This means that percentages for this category might sum more than 100%

Regarding forms of evidence, the share of participants was similar in both rounds. More than 80% of participants declared themselves as experts in evidence syntheses in both rounds, and approximately 60% reported having expertise in guidelines. The number of participants that identified themselves as having expertise in data analytics, modelling or technology assessments was reduced in the second round. All forms of evidence were represented with at least one participant in both rounds.

In round 1, the number of answers received for each type of the 40 different questions varied from 3 to 10, while the response rate varied from 13% to 83%. In round 2, the number of answers received varied from 6 to 8, while the response rate was consistently around 15% per question.

Table 2 shows the specific response rate for each type of question and the values of the Kendall’s Ws reached in the first or second round of the Delphi process, for the types of question in which a consensus was reached. In 28 types of question (out of 40), there was some evidence of consensus among the answers (Kendall’s W > 0.6 and its p-value < 0.05) reached in either round 1 or 2. S1 Table presents details of the Kendall’s W for each round, and the rank correlation (calculated from the Kendall’s W), and for participants per question (including questions in which a consensus was not reached).

**Table 2:** Response rate and Kendall’s W for the types of question in which consensus was reached.

Stage	Goal	Type of question	N round 1 (response rate)	N round 2 (response rate)	KW reached
**I. Clarifying a problem**	Choosing and prioritizing outcomes of a problem	Identifying outcomes to characterize a problem	7 (37%)	8 (18%)	0.78
		Prioritizing outcomes to characterize a problem	5 (26%)	8 (18%)	0.70
	Describing a problem and its magnitude	Describing a problem in a point in time	3 (13%)	NA	0.70
		Clarifying and characterizing populations affected by a problem	4 (17%)	NA	0.71
	Understanding a problem	Understanding stakeholders’ perceptions of a problem	9 (41%)	8 (18%)	0.66
	Assessing the variability of a problem	Assessing variability over time	3 (14%)	7 (16%)	0.80
	Understanding the impacts of a problem	Identifying impacts/spillover effects of a problem	6 (22%)	NA	0.96
		Prioritizing the most important impacts/spillover effects of a problem	6 (22%)	NA	0.90
**II. Finding options**	Finding and understanding potential options.	Scoping a list of potential options	4 (27%)	NA	0.76
		Understanding the way potential options and their components work	4 (27%)	7 (16%)	0.66
	Assessing the expected impact of options	Assessing the feasibility of an option	10 (28%)	7 (16%)	0.68
		Assessing the benefits and early-and-frequently occurring harms of an option	10 (28%)	6 (13%)	0.94
		Identifying late-occurring harms and risks of an option	10 (28%)	7 (16%)	0.72
		Assessing the costs and resource use of an option	10 (28%)	7 (16%)	0.73
		Assessing the efficiency in the use of resources	7 (19%)	NA	0.80
		Identifying equity, ethical, social and human rights impacts of an option	10 (28%)	7 (16%)	0.79
		Assessing the acceptability of an option	10 (28%)	7 (16%)	0.81
	Maximizing the expected impact of options	Finding population groups, settings and contexts to focusing options	7 (21%)	7 (16%)	0.62
	Contributing to prioritize and select options	Creating packages of options	7 (25%)	7 (16%)	0.76
		Creating a ranking of options	7 (25%)	NA	0.61
**III. Implementing AN option**	Setting up a sustainable implementation process by identifying barriers, facilitators and implementation strategies	Identifying and understanding barriers and implementation strategies to deal with them	6 (38%)	7 (16%)	0.60
	Planning and describing the implementation of an option	Identifying who has to do what to implement an option	5 (83%)	7 (16%)	0.84
		Identifying the context in which the option could be implemented	4 (67%)	7 (16%)	0.75
		Describing whether implementation of an option is underway and at what stage level	4 (67%)	7 (16%)	0.72
**IV. Monitoring and evaluation**	Identifying measurement strategies for populations and outcomes	Identifying instruments to ascertain populations	4 (24%)	6 (13%)	0.67
		Identifying measurement instruments for outcomes of interest	4 (24%)	6 (13%)	0.71
	Monitoring and evaluating populations and outcomes of interests	Measuring the impact of an option or implementation strategy	6 (27%)	NA	0.63
		Interpreting the findings of measuring the impact of an option or implementation strategy	5 (23%)	6 (13%)	0.84

KW: Kendall’s W; NA: Not available (question not included in the second round)

From the original list of types of question, because their Kendall’s W was greater than 0.6 and were statistically significant in the first round (i.e., there was consensus), 8 original questions were not moved to the second round. Because there was agreement that their ranking was last, a number of study designs were removed from the first round. One new study design suggested by a participant was added in the second round.

Tables 3 to 6 show the first four study designs in which a consensus was reached to address each one of the 28 types of question. Tables are structured depending on the specific decision-making stage in which they were classified in the original taxonomy of types of question. S2 Table shows the full ranking of study designs for all the 40 questions (including the questions in which a consensus was not reached).

**Table 3:** Ranking of the first four study designs to address the types of question in which consensus was reached in stage 1.

Goal	Type of question	Study design	Median rank (IQR)
Choosing and prioritizing outcomes of a problem	Identifying outcomes to characterize a problem	Review of outcomes that have been used by other studies (e.g., scoping review)	1 (1, 1)
		Cross-sectional study (survey, point-in-time or snapshot study or analysis) of people’s experience	2 (2, 4)
		Cross-sectional study (survey, point-in-time or snapshot study or analysis) of experts’ opinion	3 (3, 3)
	Prioritizing outcomes to characterize a problem	Delphi study (to get consensus from experts)	1 (1, 1)
		Cross-sectional study (survey, point-in-time or snapshot study or analysis) of data collected for this purpose (i.e., primary data)	2 (2, 2.25)
		Jurisdictional scan (comparative analysis) to understand what other jurisdictions are using	5 (2.75, 6)
		Cross-sectional study (survey, point-in-time or snapshot study or analysis) of data collected for other purposes (i.e., secondary data)	3.5 (3, 5.25)
Describing a problem and its magnitude	Describing a problem during a period of time	Prospective cohort study of individual-level data (prospective longitudinal or panel study)	1.5 (1, 2.25)
		Retrospective cohort study of individual-level data (retrospective or historical longitudinal, or panel study)	2 (1.75, 2.25)
		Cross-sectional study (survey, point-in-time or snapshot study or analysis) of data collected for this purpose (i.e., primary data)	3 (2.5, 3.25)
		Delphi study (to get consensus from experts)	6.5 (5.38, 6.63)
Understanding a problem	Finding conceptual approaches to understand a problem	Review to identify existing frameworks (conceptual analysis)	1 (1, 1)
		Qualitative inductive (from particular to general i.e., creating theory) methods to interpret/critically analyze a phenomenon (e.g., ethnographic approaches, phenomenology)	2 (2, 3.25)
		Review to build a new framework (critical interpretive synthesis)	3 (2.75, 3)
		Qualitative inductive (from particular to general i.e., creating theory) methods to describe a phenomenon (e.g., grounded theory)	4 (4, 4)
	Understanding stakeholders’ perceptions of a problem	Qualitative inductive (from particular to general i.e., creating theory) methods to interpret/critically analyze a phenomenon (e.g., ethnographic approaches, phenomenology)	1 (1, 1.5)
		Cross-sectional study (survey, point-in-time or snapshot study or analysis) of people’s experiences (not asking about hypothetical scenarios)	2 (2, 2)
		Qualitative inductive (from particular to general i.e., creating theory) methods to describe a phenomenon (e.g., grounded theory)	3 (3, 3.25)
		Qualitative deductive (from general to particular i.e., testing theory) methods to describe/critically analyze a phenomenon (e.g., qualitative case studies)	4 (4, 4.25)
Assessing the variability of a problem	Assessing variability over time	Retrospective cohort study of individual-level data (retrospective or historical longitudinal, or panel study)	1 (1, 1)
		Descriptive (not predicting) time-series analysis (including trend analysis)	2 (2, 2)
		Modelling to predict future scenarios (e.g., system dynamics, ARIMA models, etc.)	3 (3, 4.5)
		Single before-and-after study of aggregated data (pre-post or pretest-posttest study)	4 (3.25, 4)
		Case reports (case series)	5 (5, 5.75)
Understanding the impacts of a problem	Identifying impacts/spillover effects of a problem	Controlled before-and-after study of aggregated data (including difference-in-differences study and non-equivalent control group designs)	3.5 (1.5, 7.75)
		Review to find causes or aggravating factors that have been identified by other studies (e.g., scoping review)	3.5 (3, 7.75)
		Prospective cohort study of individual-level data (prospective longitudinal or panel study)	5 (1, 9)
		Retrospective cohort study of individual-level data (retrospective or historical longitudinal, or panel study)	6.5 (2.5, 9)
	Prioritizing the most important impacts/spillover effects of a problem	Review to find causes or aggravating factors that have been identified by other studies (e.g., scoping review)	4.5 (3.25, 6.5)
		Delphi studies (to get consensus from experts)	5.5 (1, 10.38)
		Prospective cohort study of individual-level data (prospective longitudinal or panel study)	7.25 (2, 9.88)
		Retrospective cohort study of individual-level data (retrospective or historical longitudinal, or panel study)	7.75 (3, 9.88)

**Table 4:** Ranking of the first four study designs to address the types of question in which consensus was reached in stage 2.

Goal	Type of question	Study design	Median rank (IQR)
Finding and understanding potential options.	Scoping a list of potential options	Review to find options that have been used by other studies (e.g., scoping review)	1 (1, 1)
		Jurisdictional scan (comparative analysis) to understand what options have been implemented by other jurisdictions	2 (2, 2.25)
		Cross-sectional study (survey, point-in-time or snapshot study or analysis) of people’s opinions	3.5 (2.75, 4.25)
		Ecological study (population-based study, including spatial analysis)	3.75 (3, 4.63)
	Understanding the way potential options and their components work	Randomized-controlled study (randomized experiment or randomized trial) measuring intermediate outcomes	1 (1, 3.5)
		Review to identify existing frameworks (conceptual analysis) that explain how an intervention might work	2 (1, 2)
		Interrupted time-series analysis (including joint-point regression) measuring intermediate outcomes	3 (3, 4.5)
		Descriptive (non-qualitative) case study	4 (4, 4)
Assessing the expected impact of options	Assessing the feasibility of an option	Delphi studies (to get consensus from experts)	1 (1, 2)
		Jurisdictional scan (comparative analysis) to understand the feasibility of the option elsewhere	2 (1, 2)
		Discrete choice experiment (stated preferences)	3 (3, 5.75)
		Modelling to predict whether the option will be feasible (e.g., system dynamics, ARIMA models, etc.)	4 (4, 6.75)
	Assessing the benefits and early-and-frequently occurring harms of an option	Randomized-controlled study (randomized experiment or randomized trial)	1 (1, 1)
		Controlled before-and-after study of aggregated data (including difference-in-differences study and non-equivalent control group designs)	2 (2, 2)
		Interrupted time-series analysis (including joint-point regression)	3 (3, 3)
		Retrospective cohort study of individual-level data (retrospective or historical longitudinal, or panel study)	4 (4, 5.5)
	Assessing late-occurring harms and risks of an option	Retrospective cohort study of individual-level data (retrospective or historical longitudinal, or panel study), including databases of adverse event reporting (e.g., pharmacovigilance)	1 (1, 3.5)
		Randomized-controlled study (randomized experiment or randomized trial)	2 (1, 2)
		Prospective cohort study of individual-level data (prospective longitudinal or panel study)	2 (2, 3)
		Case-control study (case-comparison study)	4 (4, 5.5)
	Assessing the costs and resource use of an option	Modelling to estimate the cost of an option	1 (1, 1)
		Jurisdictional scan (comparative analysis) to understand the costs in other jurisdictions	2 (2, 3)
		Case reports (case series)	3 (2, 7)
		Cross-sectional study (survey, point-in-time or snapshot study or analysis) of data collected for this purpose (i.e., primary data)	4 (4, 6)
	Assessing the efficiency in the use of resources	Economic evaluations (cost-effectiveness, cost-utility, cost-benefit analyses)	1 (1, 1)
		Jurisdictional scan (comparative analysis) to understand whether the option was efficient in other jurisdictions	2 (2, 2.5)
		Delphi studies (to get consensus from experts)	3 (2.5, 3)
	Identifying equity, ethical, social and human rights impacts of an option	Delphi studies (to get consensus from experts)	1 (1, 1.5)
		Cross-sectional study (survey, point-in-time or snapshot study or analysis) of people’s experiences (not asking about hypothetical scenarios)	2 (2, 2)
		Qualitative deductive (from general to particular i.e., testing theory) methods to describe/critically analyze a phenomenon (e.g., qualitative case studies)	3 (2.5, 3)
		Qualitative deductive (from general to particular i.e., testing theory) methods to describe a phenomenon (e.g., qualitative description, narrative approaches)	4 (4, 4)
	Assessing the acceptability of an option	Discrete choice experiment (stated preferences)	1 (1, 1.5)
		Qualitative deductive (from general to particular i.e., testing theory) methods to describe/critically analyze a phenomenon (e.g., qualitative case studies)	2 (2, 2)
		Qualitative deductive (from general to particular i.e., testing theory) methods to describe a phenomenon (e.g., qualitative description, narrative approaches)	3 (3, 3)
		Cross-sectional study (survey, point-in-time or snapshot study or analysis) of people’s experiences (not asking about hypothetical scenarios)	4 (4, 4)
Maximizing the expected impact of options	Finding population groups, settings and contexts to focusing options	Case-control study (case-comparison study)	1.5 (1, 2.75)
		Prospective cohort study of individual-level data (prospective longitudinal or panel study)	2 (1.25, 2)
		Controlled before-and-after study of aggregated data (including difference-in-differences study and non-equivalent control group designs)	3 (3, 5.25)
		Randomized-controlled study (randomized experiment or randomized trial) using subgroup comparisons.	4 (2.5, 4)
Contributing to prioritize and select options	Creating packages of options	Evidence synthesis of studies evaluating the impact of single interventions to analyze the combined effect of packages.	1 (1, 1)
		Randomized-controlled study (randomized experiment or randomized trial) to compare packages of interventions in different arms	2 (2, 2)
		Randomized-controlled study (randomized experiment or randomized trial) using posthoc comparisons.	3 (3, 3)
		Delphi study (to get consensus from experts)	4 (4, 4)
	Creating a ranking of options	Economic evaluations (cost-effectiveness, cost-utility, cost-benefit analyses) to create a ranked list of options	1 (1, 2)
		Ranking type Delphi study (to get consensus from experts)	2 (2, 2.5)
		Multi-criteria (objective) decision analysis to create a ranked list of options	2 (2, 3)
		Discrete choice experiment (stated preferences)	4 (4, 4.75)

**Table 5:** Ranking of the first four study designs to address the types of question in which consensus was reached in stage 3.

Goal	Type of question	Study design	Median rank (IQR)
Setting up a sustainable implementation process by identifying barriers, facilitators and implementation strategies	Identifying and understanding barriers and implementation strategies to deal with them	Review to find barriers and implementation strategies that have been used by other studies (e.g., scoping review)	1 (1, 1)
		Cross-sectional study (survey, point-in-time or snapshot study or analysis) of people’s experiences	2 (2, 2.5)
		Qualitative deductive (from general to particular i.e., testing theory) methods to describe/critically analyze a phenomenon (e.g., qualitative case studies)	4 (3.5, 4.75)
		Social network analysis (mapping network analysis) to identify barriers and implementation strategies	5 (3, 5.75)
Planning and describing the implementation of an option	Identifying who has to do what to implement an option	Delphi studies (to get consensus from experts)	1 (1, 1)
		Cross-sectional study (survey, point-in-time or snapshot study or analysis) of people’s experiences	2 (2, 2)
		Qualitative inductive (from particular to general i.e., creating theory) methods to describe a phenomenon (e.g., grounded theory)	3 (3, 4.75)
		Qualitative deductive (from general to particular i.e., testing theory) methods to describe a phenomenon (e.g., qualitative description, narrative approaches)	4 (4, 4)
	Identifying the context in which the option could be implemented	Jurisdictional scan (comparative analysis) to understand what other jurisdictions have identified as contextual variables	1 (1, 1)
		Qualitative deductive (from general to particular i.e., testing theory) methods to describe a phenomenon (e.g., qualitative description, narrative approaches)	2 (2, 3.5)
		Cross-sectional study (survey, point-in-time or snapshot study or analysis) of people’s experiences	3 (3, 3.5)
		Delphi studies (to get consensus from experts)	4 (3, 4)
	Describing whether implementation of an option is underway and at what stage level	Cross-sectional study (survey, point-in-time or snapshot study or analysis) of people’s experiences	1 (1, 1)
		Descriptive (not predicting) time-series analysis (including trend analysis)	2 (2, 3.75)
		Qualitative deductive (from general to particular i.e., testing theory) methods to describe/critically analyze a phenomenon (e.g., qualitative case studies)	3 (2.5, 3)
		Qualitative deductive (from general to particular i.e., testing theory) methods to describe a phenomenon (e.g., qualitative description, narrative approaches, documentary review of public speeches, etc.)	4 (4, 4.75)

**Table 6:** Ranking of the first four study designs to address the types of question in which consensus was reached in stage 4.

Goal	Type of question	Study design	Median rank (IQR)
Identifying measurement strategies for populations and outcomes	Identifying instruments to ascertain populations	Review to find measurement strategies that have been used by other studies (e.g., scoping review)	1 (1, 3)
		Cross-sectional study (survey, point-in-time or snapshot study or analysis) of people’s opinions on measurement strategies	2 (1, 3)
		Jurisdictional scan (comparative analysis) to understand what measurement strategies have been used by other jurisdictions	2 (2, 2)
		Descriptive (non-qualitative) case study	4 (4, 5)
	Identifying measurement instruments for outcomes of interest	Review to find measurement strategies that have been used by other studies (e.g., scoping review)	1 (1, 1)
		Jurisdictional scan (comparative analysis) to understand what measurement strategies have been used by other jurisdictions	2 (2, 2)
		Descriptive (non-qualitative) case study	3 (3, 3.75)
		Cross-sectional study (survey, point-in-time or snapshot study or analysis) of people’s opinions on measurement strategies	4 (4, 4)
Monitoring and evaluating populations and outcomes of interests	Measuring the impact of an option or implementation strategy	Randomized-controlled study (randomized experiment or randomized trial), including pragmatic trials	1 (1, 1)
		Controlled before-and-after study of aggregated data (including difference-in-differences study and non-equivalent control group designs)	3.5 (2.25, 4)
		Interrupted time-series analysis (including joint-point regression)	3.5 (3, 4)
		Regression discontinuity study (regression kink study or analysis)	5 (2.5, 8.625)
	Interpreting the findings of measuring the impact of an option or implementation strategy	Cross-sectional study (survey, point-in-time or snapshot study or analysis) of people’s experiences (not asking about hypothetical scenarios)	1 (1, 1)
		Qualitative deductive (from general to particular i.e., testing theory) methods to describe a phenomenon (e.g., qualitative description, narrative approaches)	2 (2, 2)
		Descriptive (non-qualitative) case study	3 (3, 3)
		Qualitative deductive (from general to particular i.e., testing theory) methods to describe/critically analyze a phenomenon (e.g., qualitative case studies)	4 (4, 4)

In stage I (clarifying a societal problem, its causes, and potential impacts), shown in Table 3, 8 (out of 15) types of question reached a consensus. A clear gold standard (i.e., a study design the median rank of which was 1, and its interquartile range does not alter its ranking as first) was identified in five of the questions included, whereas in all of the types of question one or more study designs were clearly identified as a last priority (i.e., a study design whose median rank was last, and its interquartile range does not alter its ranking as last).

Stage I has most of the types of questions in which a consensus was not reached (7). These questions were related to understanding stakeholders’ values regarding outcomes, describing a problem and its magnitude at a point in time, understanding the role of the context in a given problem, assessing in the variability of the problem across populations and locations and relative to other problems, and understanding the causes and/or aggravating factors of a problem.

In stage II (finding options to address a problem), shown in Table 4, 12 (out of 13) types of question reached a consensus. A clear gold standard was clearly identified in 7 of the questions, whereas in only one of them was at least one study design not identified as clear last priority. There was only one type of question in which a consensus was not reached, which was related to adjusting options to maximize their impacts.

In stage III (implementing or scaling-up an option), shown in Table 5, four (out of six) types of questions reached a consensus. A clear gold standard was identified in all four questions included. In only one question there was not at least one study design identified as clear last priority. Two types of questions did not reach a consensus and were related to the identification and prioritization of facilitators and implementation strategies to take advantage of them.

In stage IV (monitoring implementation and evaluating impacts), shown in Table 6, four (out of six) types of questions reached a consensus. A clear gold standard was identified in three of the questions, and in two of them at least one study design was identified as clear last priority.

There were two types of questions where a consensus could not be reached. These They were related to the first goal (identifying measurement strategies for populations and outcomes). While the two other questions in this goal reached a consensus, the questions related to choosing the most accurate approach, and determining the best instruments, to measure/ascertain populations and outcomes did not reach consensus.

## Discussion

### Principal findings and findings in relation to the existing literature

This study used a two-round Delphi process to produce a list of preferred study designs that investigators can use to address 28 different types of questions that were part of a mutually exclusive and collectively exhaustive taxonomy of types of questions in which evidence could provide support (S1 Fig). To reach a consensus on preferred study designs, the study elicited opinions from 29 experts.

In stage 1 (clarifying a problem, its causes and potential impacts), five types of question had clear preferred study designs (scoping reviews to identifying outcomes; Delphi studies to prioritize outcomes; reviews of frameworks to find conceptual approaches; qualitative inductive designs to understand stakeholders’ perceptions and retrospective cohort study to assess the variability of a problem over time, while seven types of question (scoping reviews to scope a list of potential options, randomized-controlled study to assess benefits and early-and-frequently occurring harms, modelling to assess the costs, economic evaluations to assess the efficiency in the use of resources, Delphi studies to identify equity, ethical and social and human rights impacts, discrete choice experiments to assess the acceptability, and evidence syntheses to create packages of options) fill this criteria in stage 2 (finding and selecting options). In stage 3 (implementing an option), four types of question had a preferred study design (scoping reviews to identify and understand barriers, Delphi studies to identify who has to do what, jurisdictional scans to identify the context in which the option could be implemented, and cross-sectional studies to describe whether implementation is underway), while three types of question (scoping reviews to identify measurement instruments, randomized-controlled studies to measure the impact of an option, and cross-sectional study to interpret the findings of measuring the impact) fill this criteria in stage 4 (monitoring implementation and evaluating impacts).

A consensus was not reached in 12 of the 40 different types of questions, the majority of which were part of the first decision-making stage (clarifying a societal problem, its causes, and potential impacts). In the first round of the Delphi, 8 types of questions reached a consensus and were not included in the subsequent stage, while 20 reached a consensus in the second stage when they had not reached a consensus in the first stage.

Previous research efforts have generated hierarchies or guidance of evidence for some types of questions or study designs [16, 17] and includes a more pragmatic approach to build guidance on the suitability of study designs to address demand-driven types of question. In this sense, this study moves beyond a static hierarchy or guidance of study designs, assuming that different study designs might more suitably answer different types of question. Regardless of the acceptance of a hierarchy, typology or a list of study designs, it is largely accepted that the traditional hierarchy of evidence (having RCTs and systematic reviews on top of a pyramid) could only provide insights for one type of question (which is represented twice in this paper as “Assessing the benefits and early-and-frequently occurring harms of an option”, and “Measuring the impact of an option or implementation strategy”) and that different types of study designs would be more appropriate than others based on the specific type of question that is to be addressed [9, 10].

### Strengths and limitations

This paper has a number of strengths. First, it creates a list of preferred study designs that can answer specific types of questions collected using a demand-driven approach (i.e., hence, are more likely to be related to the questions that decision-makers might ask). Secondly, it does so in a way that gave voice to various methodological experts, sampling by eight different forms of evidence and WHO region. Finally, it also does so using a robust methodology to reach a strong consensus and identify critical areas in which further work is required to reach a consensus (if possible), using a two-round Delphi process conducted online to reach broader audiences.

This study also has limitations. First, Delphi studies have the potential of acquiescence bias, which is the tendency of the participants to agree with the existing result, and thus failing to address their true preference. Secondly, since we prioritized the questions each expert received based on their methodological expertise, the methodological experts answered different sets of questions included in the first round of the Delphi. However, this contributed to reaching a higher response rate, particularly in round 1. Thirdly, despite all the efforts were made to have a higher response rate, round two of the Delphi reached a low response rate. Finally, while we used Kendall’s W and rank correlation to quantify the consensus reached in ranking-type surveys, these statistical measures have potential biases, such as not properly discriminating the distance between ranks (e.g., the disagreement arising from ranking in the sixth versus seventh place is not different from the disagreement arising from ranking in the first or second place).

### Implications for policy and practice

Evidence intermediaries can use the results of this paper in their role of promoting the use of evidence in decision-making processes. By identifying the type of question that decision makers are asking, they can either search or commission a study using a design that would be well suited to address that particular need. Researchers can use the results of this paper to guide them in choosing the preferred study design for the type of question at hand. Finally, funders can use this guidance to decide what type of study design to fund or use, depending on the specific need at hand.

### Implications for future research

Future research could explore several different areas. Firstly, subsequent efforts can explore reaching a consensus on the 12 types of questions in which a consensus was not reached. While a third round of the Delphi process could have been conducted, we might have a very limited number of responses. Alternatively, we suggest conducting a series of structured meetings (that follows the approach used by the GRADE Working Group to reach a consensus [18]) in which methodological experts can discuss what approaches can be better for specific types of questions.

Secondly, future studies could explore how considerations beyond study design (e.g., data analysis for any given study design) could play a role in answering specific types of question. Thirdly, further research could weigh the role of domestic versus global evidence, and primary studies versus evidence syntheses as complementary approaches to provide an evidence-informed answer for specific types of question. Finally, while qualitative study designs were included as part of this study, they were only part of a high-level category based on their paradigm (deductive/inductive) and general aim (to describe/ to critically interpret). Future research could go beyond this and use specific qualitative study designs to understand better what type of study designs are better than others to address a given type of question.

## Supporting information

S1 Fig A demand-driven taxonomy of the types of question that could be addressed by evidence. (PDF)

S1 Table Response rates and consensus levels reached by all the questions included in the study. (DOCX)

S2 Table Complete prioritized list of study designs used to address each type of question. (DOCX)

## References

[1] Lavis JN, Wilson MG, Oxman AD, Lewin S, Fretheim A. SUPPORT Tools for evidence-informed health Policymaking (STP) 4: Using research evidence to clarify a problem. Health Research Policy and Systems. 2009;7:S4. doi: 10.1186/1478-4505-7-S1-S4 20018111 PMC3271831

[2] Lavis JN, Wilson MG, Oxman AD, Grimshaw J, Lewin S, Fretheim A. SUPPORT Tools for evidence-informed health Policymaking (STP) 5: Using research evidence to frame options to address a problem. Health Research Policy and Systems. 2009;7:S5. doi: 10.1186/1478-4505-7-S1-S5 20018112 PMC3271832

[3] Fretheim A, Munabi-Babigumira S, Oxman AD, Lavis JN, Lewin S. SUPPORT Tools for Evidence-informed Policymaking in health 6: Using research evidence to address how an option will be implemented. Health Res Policy Sys. 2009;7:S6. 10.1186/1478-4505-7-S1-S6 PMC3271833 20018113

[4] Pearson H. How COVID broke the evidence pipeline. Nature. 2021;593:182–5. doi: 10.1038/d41586-021-01246-x 33981057

[5] Cochrane Convenes. Preparing for and responding to global health emergencies: Learnings from the COVID-19 evidence response and recommendations for the future. February 2022 [Internet]. 2022. https://figshare.com/articles/book/Preparing_for_and_responding_to_global_health_emergencies_Learnings_from_the_COVID-19_evidence_response_and_recommendations_for_the_future/19115849

[6] Global Commission on Evidence to Address Societal Challenges. The Evidence Commission report: A wake-up call and path forward for decision-makers, evidence intermediaries, and impact-oriented evidence producers [Internet]. 2022 [cited 2023 Jan 4]. https://www.mcmasterforum.org/docs/default-source/evidence-commission/evidence-commission-report.pdf?Status=Master&sfvrsn=2fb92517_5/Evidence-Commission-report

[7] Kuchenmüller T, Lavis J, Kheirandish M, Reveiz L, Reinap M, Okeibunor J, et al. Time for a new global roadmap for supporting evidence into action. Robinson J, editor. PLOS Glob Public Health. 2022;2:e0000677. 36962468 10.1371/journal.pgph.0000677 PMC10022000

[8] Global Commission on Evidence to Address Societal Challenges. Evidence Commission update 2023: Strengthening domestic evidence-support systems, enhancing the global evidence architecture, and putting evidence at the centre of everyday life [Internet]. McMaster Health Forum; 2023 [cited 2023 Feb 27]. https://www.mcmasterforum.org/docs/default-source/evidence-commission/update-2023.pdf?sfvrsn=e81cbf_8

[9] Evans D. Hierarchy of evidence: a framework for ranking evidence evaluating healthcare interventions. J Clin Nurs. 2003;12:77–84. doi: 10.1046/j.1365-2702.2003.00662.x 12519253

[10] Petticrew M. Evidence hierarchies, and typologies: horses for courses. Journal of Epidemiology & Community Health. 2003;57:527–9. doi: 10.1136/jech.57.7.527 12821702 PMC1732497

[11] Agoritsas T, Vandvik PO, Neumann I, Rochwerg B, Jaeschke R, Hayward R, et al. Finding Current Best Evidence. In: Guyatt G, Rennie D, Meade MO, Cook DJ, editors. Users’ Guides to the Medical Literature: A Manual for Evidence-Based Clinical Practice, 3rd ed [Internet]. New York, NY: McGraw-Hill Education; 2015 [cited 2023 Jul 27]. jamaevidence.mhmedical.com/content.aspx?aid=1183875650

[12] Strasser A. Design and evaluation of ranking-type Delphi studies using best-worst-scaling. Technology Analysis & Strategic Management. 2019;31:492–501.

[13] Kobus J, Westner M. Ranking-type delphi studies in IS research: step-by-step guide and analytical extension. 2016.

[14] Kendall Maurice, Jean Dickinson Gibbons. Rank correlation methods. 5th Edition. Oxford University Press; 1990.

[15] Zaiontc, C. Real Statistics Using Excel [Internet]. 2020 [cited 2023 May 3]. www.real-statistics.com

[16] Parkhurst JO, Abeysinghe S. What Constitutes “Good” Evidence for Public Health and Social Policy-making? From Hierarchies to Appropriateness. Social Epistemology. 2016;30:665–79.

[17] Daly J, Willis K, Small R, Green J, Welch N, Kealy M, et al. A hierarchy of evidence for assessing qualitative health research. Journal of Clinical Epidemiology. 2007;60:43–9. doi: 10.1016/j.jclinepi.2006.03.014 17161753

[18] Guyatt GH, Oxman AD, Vist GE, Kunz R, Falck-Ytter Y, Alonso-Coello P, et al. GRADE: an emerging consensus on rating quality of evidence and strength of recommendations. BMJ. 2008;336:924–6. doi: 10.1136/bmj.39489.470347.AD 18436948 PMC2335261

