# Supplementary material for: Matching the right study design to decision-maker questions: Results from a Delphi study
Source: PLOS Glob Public Health. 2024 Feb 29;4(2):e0002752. doi: 10.1371/journal.pgph.0002752 (PMC10903833; doi:10.1371/journal.pgph.0002752)
Supplement: S1 Fig — (PDF) [file pgph.0002752.s001.pdf]

Clarifying a societal **problem**, its causes and potential impacts

Goal of question

- A. choosing and prioritizing measurements of a problem
- B. describing a problem and its magnitude
- C. understanding a problem
- D. assessing the variability of a problem
- E. understanding the causes and aggravating factors of a problem
- F. understanding the impacts of a problem

Finding and selecting **options** to address a problem

Goal of question

- A. finding and understanding potential options
- B. assessing the expected impact of options
- C. maximizing the expected impact of options
- D. contributing to prioritize and select options

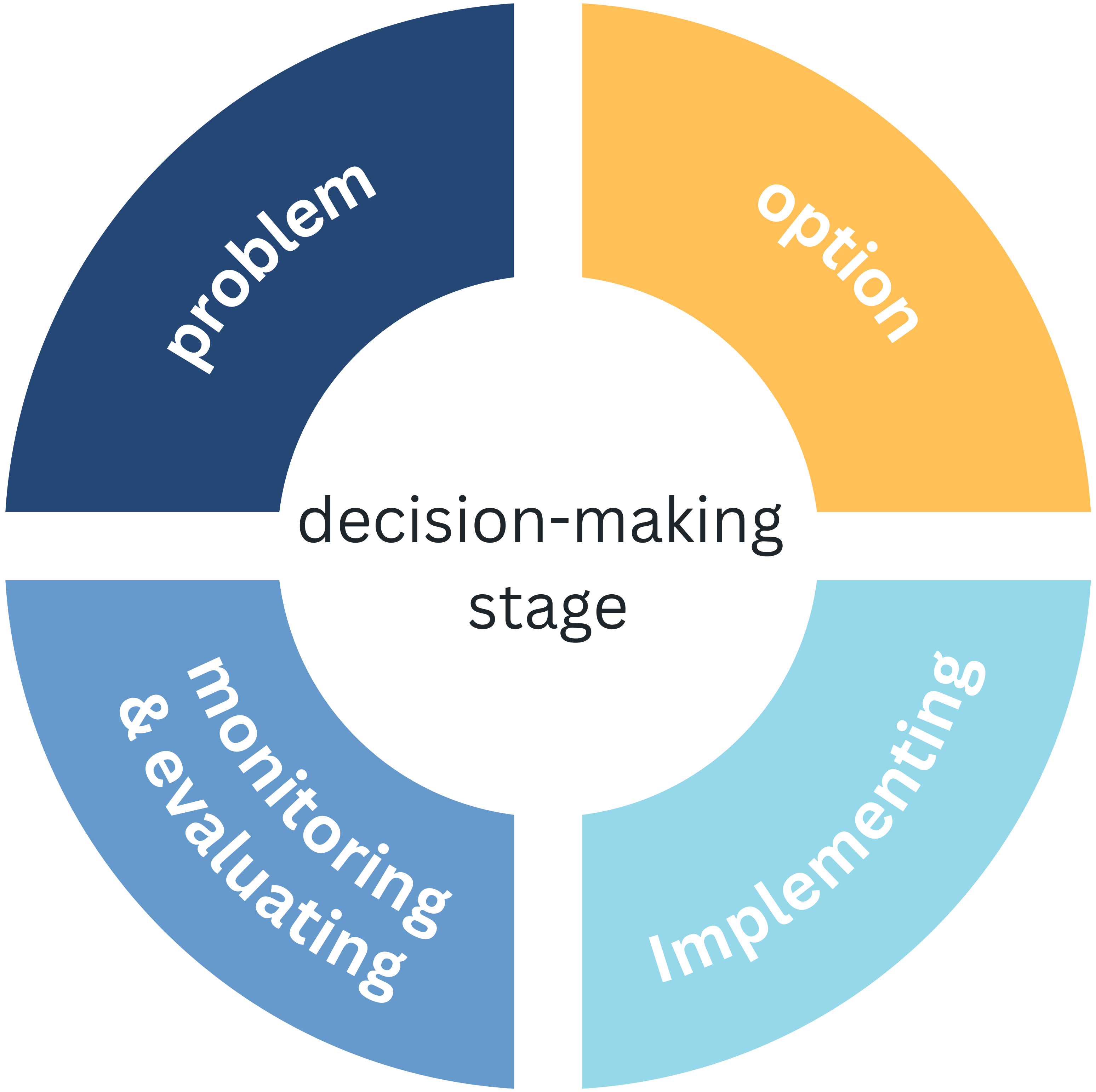

Monitoring implementation and **evaluating** impacts

Goal of question

- A. identifying measurement strategies for populations and outcomes
- B. monitoring and evaluating populations and outcomes of interest

Implementing or scaling-up an option

Goal of question

- A. planning and describing the implementation of an option
- B. setting up a sustainable implementation process by identifying barriers, facilitators and implementation strategies
