# Supplementary material for: Matching the right study design to decision-maker questions: Results from a Delphi study
Source: PLOS Glob Public Health. 2024 Feb 29;4(2):e0002752. doi: 10.1371/journal.pgph.0002752 (PMC10903833; doi:10.1371/journal.pgph.0002752)
Supplement: S1 Table — (DOCX) [file pgph.0002752.s002.docx]

**S1 Table. Response rates and consensus reached in each Delphi round for each question**

| **Stage** | **Goal** | **Type of question** | **Round 1** | | | **Round 2** | | | |
| --- | --- | --- | --- | --- | --- | --- | --- | --- | --- |
|  |  |  | **N**  **(response rate)** | **KW**** | **RC***** | **N**  **(response rate)** | **% of agree with last rank** | **KW**** | **RC***** |
| **I. Clarifying a problem** | A. Choosing and prioritizing outcomes of a problem | 1. Identifying outcomes to characterize a problem | 7 (36.8%) | 0.30 | 0.18 | 8 (17.8%) | 63% | 0.78* | 0.75 |
|  |  | 2. Understanding individuals' values regarding outcomes | 6 (31.6%) | 0.16 | -0.01 | 8 (17.8%) | 38% | 0.45* | 0.37 |
|  |  | 3. Prioritizing outcomes to characterize a problem | 5 (26.3%) | NA | NA | 8 (17.8%) | 50% | 0.70* | 0.65 |
|  | B. Describing a problem and its magnitude | 1. Describing a problem in a point in time | 3 (12.5%) | 0.70* | 0.55 |  |  |  |  |
|  |  | 2. Clarifying and characterizing populations affected by a problem | 4 (16.7%) | 0.71* | 0.62 |  |  |  |  |
|  | C. Understanding a problem | 1. Finding conceptual approaches to understand a problem | 10 (45.5%) | 0.43* | 0.36 | 8 (17.8%) | 63% | 0.53* | 0.46 |
|  |  | 2. Understanding stakeholders' perceptions of a problem | 9 (40.9%) | 0.16 | 0.05 | 8 (17.8%) | 63% | 0.66* | 0.61 |
|  |  | 3. Understanding the role of context in a problem | 9 (40.9%) | 0.20 | 0.10 | 8 (17.8%) | 38% | 0.29* | 0.18 |
|  | D. Assessing the variability of a problem | 1. Assessing variability over time | 3 (14.3%) | 0.17 | -0.25 | 7 (15.6%) | 43% | 0.80* | 0.77 |
|  |  | 2. Assessing variability across populations and locations | 4 (19%) | NA | NA | 7 (15.6%) | 43% | 0.49* | 0.40 |
|  |  | 3. Assessing the importance of a problem relative to other problems | 4 (19%) | 0.43 | 0.24 | 7 (15.6%) | 43% | 0.52* | 0.44 |
|  | E. Understanding the causes and aggravating factors of a problem | 1. Identifying causes and/or aggravating factors of a problem | 5 (22.7%) | NA | NA | 7 (15.6%) | 29% | 0.52* | 0.45 |
|  |  | 2. Understanding the relative importance of causes and/or aggravating factors across population groups | 5 (22.7%) | NA | NA | 6 (13.3%) | 33% | 0.45* | 0.34 |
|  | F. Understanding the impacts of a problem | 1. Identifying impacts/spillover effects of a problem | 6 (22.2%) | 0.96* | 0.96 |  |  |  |  |
|  |  | 2. Prioritizing the most important impacts/spillover effects of a problem | 6 (22.2%) | 0.90* | 0.88 |  |  |  |  |
| **II. Finding options** | A. Finding and understanding potential options. | 1. Scoping a list of potential options | 4 (26.7%) | 0.76* | 0.68 |  |  |  |  |
|  |  | 2. Understanding the way potential options and their components work | 4 (26.7%) | NA | NA | 7 (15.6%) | 57% | 0.66* | 0.61 |
|  | B. Assessing the expected impact of options | 1. Assessing the feasibility of an option | 10 (27.8%) | 0.08 | -0.02 | 7 (15.6%) | 43% | 0.68* | 0.62 |
|  |  | 2. Assessing the benefits and early-and-frequently ocurring harms of an option | 10 (27.8%) | 0.26* | 0.18 | 6 (13.3%) | 50% | 0.94* | 0.93 |
|  |  | 3. Identifying late-ocurring harms and risks of an option | 10 (27.8%) | 0.13 | 0.03 | 7 (15.6%) | 43% | 0.72* | 0.68 |
|  |  | 4. Assessing the costs and resource use of an option | 10 (27.8%) | 0.26* | 0.18 | 7 (15.6%) | 29% | 0.73* | 0.69 |
|  |  | 5. Assessing the efficiency in the use of resources | 7 (19.4%) | 0.80* | 0.76 |  |  |  |  |
|  |  | 6. Identifying equity, ethical, social and human rights impacts of an option | 10 (27.8%) | 0.30* | 0.22 | 7 (15.6%) | 71% | 0.79* | 0.76 |
|  |  | 7. Assessing the acceptability of an option | 10 (27.8%) | 0.15 | 0.06 | 7 (15.6%) | 71% | 0.81* | 0.78 |
|  | C. Maximizing the expected impact of options | 1. Adjusting options and enabling factors to maximize impact | 8 (24.2%) | 0.20 | 0.09 | 7 (15.6%) | 43% | 0.59* | 0.53 |
|  |  | 2. Finding population groups, settings and contexts to focusing options | 7 (21.2%) | 0.11 | -0.03 | 7 (15.6%) | 43% | 0.62* | 0.56 |
|  | D. Contributing to prioritize and select options | 1. Creating packages of options | 7 (25%) | 0.51* | 0.42 | 7 (15.6%) | 71% | 0.76* | 0.72 |
|  |  | 2. Creating a ranking of options | 7 (25%) | 0.61* | 0.55 |  |  |  |  |
| **III. Implementnig options** | A. Setting up a sustainable implementation process by identifying barriers, facilitators and implementation strategies | 1. Identifying and understanding barriers and implementation strategies to deal with them | 6 (37.5%) | 0.33* | 0.19 | 7 (15.6%) | 43% | 0.60* | 0.53 |
|  |  | 2. Identifying and understanding facilitators and implementation strategies to take advantage of them | 6 (37.5%) | 0.38* | 0.26 | 7 (15.6%) | 43% | 0.50* | 0.41 |
|  |  | 3. Prioritizing barriers, facilitators and implementation strategies | 6 (37.5%) | 0.40* | 0.28 | 7 (15.6%) | 43% | 0.51* | 0.43 |
|  | B. Planning and describing the implementation of an option | 1. Identifying who has to do what to implement an option | 5 (83.3%) | 0.27 | 0.08 | 7 (15.6%) | 71% | 0.84* | 0.81 |
|  |  | 2. Identifying the context in which the option could be implemented | 4 (66.7%) | 0.34 | 0.12 | 7 (15.6%) | 43% | 0.75* | 0.71 |
|  |  | 3. Describing whether implementation of an option is underway and at what stage level | 4 (66.7%) | 0.30 | 0.06 | 7 (15.6%) | 43% | 0.72* | 0.67 |
| **IV. Monitoring and evaluation** | A. Identifying measurement strategies for populations and outcomes | 1. Identifying instruments to ascertain populations | 4 (23.5%) | 0.24 | -0.02 | 6 (13.3%) | 33% | 0.67* | 0.61 |
|  |  | 2. Choosing the most accurate instruments to ascertain populations | 5 (29.4%) | 0.25 | 0.07 | 6 (13.3%) | 50% | 0.53* | 0.44 |
|  |  | 3. Identifying measurement instruments for outcomes of interest | 4 (23.5%) | 0.39 | 0.18 | 6 (13.3%) | 67% | 0.71* | 0.65 |
|  |  | 4. Determining the best instruments to measure outcomes of interest | 5 (29.4%) | 0.35 | 0.19 | 6 (13.3%) | 33% | 0.54* | 0.45 |
|  | B. Monitoring and evaluating populations and outcomes of interests | 1. Measuring the impact of an option or implementation strategy | 6 (27.3%) | 0.63* | 0.56 |  |  |  |  |
|  |  | 2. Interpreting the findings of measuring the impact of an option or implementation strategy | 5 (22.7%) | 0.30 | 0.12 | 6 (13.3%) | 67% | 0.84* | 0.808 |

*: p-value < 0.05

**KW: Kendall’s W. Values closer to one means higher consensus rate.

***RC: Spearman rank correlation; Values closer to 1 means higher consensus rate.

NA: Not available (the first round produced too many ties to calculate a valid Kendall’s W); Blank cells are questions that were not included in the second round.
