# Supplementary material for: Matching the right study design to decision-maker questions: Results from a Delphi study
Source: PLOS Glob Public Health. 2024 Feb 29;4(2):e0002752. doi: 10.1371/journal.pgph.0002752 (PMC10903833; doi:10.1371/journal.pgph.0002752)
Supplement: S2 Table — (DOCX) [file pgph.0002752.s003.docx]

**S2 Table A. Ranking of study designs per Delphi round in stage I. Clarifying a societal problem, its causes and potential impacts**

| **Goal** | **Type of question** | **Study design** | **Median rank (IQR) round 1** | **Median rank (IQR) round 2** |
| --- | --- | --- | --- | --- |
| A. Choosing and prioritizing outcomes of a problem | 1. Identifying outcomes to characterize a problem | Review of outcomes that have been used by other studies (e.g., scoping review) | 1(1,1) | 1(1,1) |
|  |  | Cross-sectional study (survey, point-in-time or snapshot study or analysis) of people’s experience | 3(3,4) | 2(2,4) |
|  |  | Cross-sectional study (survey, point-in-time or snapshot study or analysis) of experts' opinion | 3(2.5,4) | 3(3,3) |
|  |  | Jurisdictional scan (comparative analysis) to understand what other jurisdictions are currently using | 4(2.5,5.5) | 4(2.75,4) |
|  |  | Qualitative deductive (from general to particular i.e., testing theory) methods to describe a phenomenon (e.g., qualitative description, narrative approaches) | 5(3,5.5) | 5(5,5) |
|  |  | Qualitative inductive (from particular to general i.e., creating theory) methods to describe a phenomenon (e.g., grounded theory) | 5.5(3,5.75) | 6(5.875,6) |
|  | 2. Understanding individuals' values regarding outcomes | Cross-sectional study (survey, point-in-time or snapshot study or analysis) of people’s experiences (not asking about hypothetical scenarios) | 2.5(1.25,3) | 1(1,1.25) |
|  |  | Discrete choice experiment (stated preferences) | 3.5(1.5,5.125) | 2(2,4.625) |
|  |  | Qualitative inductive (from particular to general i.e., creating theory) methods to interpret/critically analyze a phenomenon (e.g., ethnographic approaches, phenomenology) | 4(3.25,5.125) | 4(2.75,4.25) |
|  |  | Case reports (case series) of people’s experience | 3.5(2.25,4) | 4(3,4.625) |
|  |  | Qualitative deductive (from general to particular i.e., testing theory) methods to describe/critically analyze a phenomenon (e.g., qualitative case studies) | 4.5(2.5,5.375) | 5(4.375,5) |
|  |  | Qualitative deductive (from general to particular i.e., testing theory) methods to describe a phenomenon (e.g., qualitative description, narrative approaches) | 5.5(2.875,5.5) | 5.25(3,6) |
|  | 3. Prioritizing outcomes to characterize a problem | Delphi study (to get consensus from experts) | 2(1,2) | 1(1,1) |
|  |  | Cross-sectional study (survey, point-in-time or snapshot study or analysis) of data collected for this purpose (i.e., primary data) | 8(3,8) | 2(2,2.25) |
|  |  | Jurisdictional scan (comparative analysis) to understand what other jurisdictions are using | 8(3,8) | 5(2.75,6) |
|  |  | Cross-sectional study (survey, point-in-time or snapshot study or analysis) of data collected for other purposes (i.e., secondary data) | 8(4,8) | 3.5(3,5.25) |
|  |  | Discrete choice experiment (stated preferences) | 8(8,8) | 5(4,6.25) |
|  |  | Ecological study (population-based study, including spatial analysis) | 8(4,8) | 5.5(5,7.125) |
|  |  | Qualitative deductive (from general to particular i.e., testing theory) methods to describe/critically analyze a phenomenon (e.g., qualitative case studies) | 8(3,8) | 7(4.75,8) |
|  |  | Modelling to compare different measurements | 8(4,8) | 7(7,7) |
|  |  | Nominal group technique (NGT) | NA | 8.5(7.375,9) |
|  |  | Prospective cohort study of individual-level data (prospective longitudinal or panel study) | 8(8,8) | NA |
|  |  | Retrospective cohort study of individual-level data (retrospective or historical longitudinal, or panel study) | 8(8,8) | NA |
|  |  | Multi-criteria (objective) decision analysis | 12(12,12) | NA |
|  |  | Qualitative deductive (from general to particular i.e., testing theory) methods to describe a phenomenon (e.g., qualitative description, narrative approaches) | 12(12,12) | NA |
| B. Describing a problem and its magnitude | 1. Describing a problem in a point in time | Cross-sectional study (survey, point-in-time or snapshot study or analysis) of data collected for this purpose (i.e., primary data) | 1(1,1.5) | NA |
|  |  | Cross-sectional study (survey, point-in-time or snapshot study or analysis) of data collected for other purposes (i.e., secondary data) | 3(2.5,3) | NA |
|  |  | Delphi study (to get consensus from experts) | 4(2.5,5) | NA |
|  |  | Case reports (case series) | 6(5,6) | NA |
|  |  | Jurisdictional scan (comparative analysis) to understand what other jurisdictions are using | 6(4,6) | NA |
|  |  | Prospective cohort study of individual-level data (prospective longitudinal or panel study) | 6(5,6) | NA |
|  |  | Retrospective cohort study of individual-level data (retrospective or historical longitudinal, or panel study) | 6(4.5,6) | NA |
|  |  | Modelling to estimate a reality or an indicator | 8(8,8) | NA |
|  | 2. Describing a problem during a period of time | Prospective cohort study of individual-level data (prospective longitudinal or panel study) | 1.5(1,2.25) | NA |
|  |  | Retrospective cohort study of individual-level data (retrospective or historical longitudinal, or panel study) | 2(1.75,2.25) | NA |
|  |  | Cross-sectional study (survey, point-in-time or snapshot study or analysis) of data collected for this purpose (i.e., primary data) | 3(2.5,3.25) | NA |
|  |  | Delphi study (to get consensus from experts) | 6.5(5.375,6.625) | NA |
|  |  | Jurisdictional scan (comparative analysis) to understand what other jurisdictions are using | 6.5(5.875,6.625) | NA |
|  |  | Cross-sectional study (survey, point-in-time or snapshot study or analysis) of data collected for other purposes (i.e., secondary data) | 6.5(5.875,6.625) | NA |
|  |  | Case reports (case series) | 6.5(6.125,6.5) | NA |
|  |  | Modelling to estimate a reality or an indicator | 6.5(5.875,6.5) | NA |
| C. Understanding a problem | 1. Finding conceptual approaches to understand a problem | Review to identify existing frameworks (conceptual analysis) | 1(1,3) | 1(1,1) |
|  |  | Qualitative inductive (from particular to general i.e., creating theory) methods to interpret/critically analyze a phenomenon (e.g., ethnographic approaches, phenomenology) | 2(2,3.75) | 2(2,3.25) |
|  |  | Review to build a new framework (critical interpretive synthesis) | 2.5(2,4.75) | 3(2.75,3) |
|  |  | Qualitative inductive (from particular to general i.e., creating theory) methods to describe a phenomenon (e.g., grounded theory) | 3(1.5,3) | 4(4,4) |
|  |  | Descriptive (non-qualitative) case study | 5(4.125,5) | 5(4.5,5) |
|  | 2. Understanding stakeholders' perceptions of a problem | Qualitative inductive (from particular to general i.e., creating theory) methods to interpret/critically analyze a phenomenon (e.g., ethnographic approaches, phenomenology) | 2(2,3) | 1(1,1.5) |
|  |  | Cross-sectional study (survey, point-in-time or snapshot study or analysis) of people's experiences (not asking about hypothetical scenarios) | 3(1,5.5) | 2(2,2) |
|  |  | Qualitative inductive (from particular to general i.e., creating theory) methods to describe a phenomenon (e.g., grounded theory) | 4(2,5.5) | 3(3,3.25) |
|  |  | Qualitative deductive (from general to particular i.e., testing theory) methods to describe/critically analyze a phenomenon (e.g., qualitative case studies) | 4(2,5) | 4(4,4.25) |
|  |  | Qualitative deductive (from general to particular i.e., testing theory) methods to describe a phenomenon (e.g., qualitative description, narrative approaches) | 4(3,5) | 5(4.5,5) |
|  |  | Discrete choice experiment (stated preferences) | 5(3,5.5) | 6(5.75,6) |
|  | 3. Understanding the role of context in a problem | Qualitative inductive (from particular to general i.e., creating theory) methods to interpret/critically analyze a phenomenon (e.g., ethnographic approaches, phenomenology) | 2(1,3) | 1.5(1,3) |
|  |  | Jurisdictional scan (comparative analysis) to understand the role that the context had in other jurisdictions | 6(3,6) | 3.5(1,5) |
|  |  | Qualitative inductive (from particular to general i.e., creating theory) methods to describe a phenomenon (e.g., grounded theory) | 4(2,6) | 2.5(2,5.125) |
|  |  | Qualitative deductive (from general to particular i.e., testing theory) methods to describe a phenomenon (e.g., qualitative description, narrative approaches) | 5(3,6) | 3.5(3,5.625) |
|  |  | Social network analysis (mapping network analysis) | 5(3,6.5) | 4(4,5.625) |
|  |  | Cross-sectional study (survey, point-in-time or snapshot study or analysis) of people's experiences | 6(4,7) | 5.5(3.75,6) |
|  |  | Qualitative deductive (from general to particular i.e., testing theory) methods to describe/critically analyze a phenomenon (e.g., qualitative case studies) | 6(2,6.5) | 6.75(5,7) |
|  |  | Descriptive (non-qualitative) case study | 6.5(6,6.5) | NA |
| D. Assessing the variability of a problem | 1. Assessing variability over time | Retrospective cohort study of individual-level data (retrospective or historical longitudinal, or panel study) | 2(1.5,3.75) | 1(1,1) |
|  |  | Descriptive (not predicting) time-series analysis (including trend analysis) | 3(2,3) | 2(2,2) |
|  |  | Modelling to predict future scenarios (e.g., system dynamics, ARIMA models, etc.) | 4(3,4.75) | 3(3,4.5) |
|  |  | Single before-and-after study of aggregated data (pre-post or pretest-posttest study) | 4(3.5,4) | 4(3.25,4) |
|  |  | Case reports (case series) | 5.5(3.25,5.5) | 5(5,5.75) |
|  |  | Prospective cohort study of individual-level data (prospective longitudinal or panel study) | 5.5(3.75,5.5) | 5.75(4.375,6) |
|  | 2. Assessing variability across populations and locations | Cross-sectional study (survey, point-in-time or snapshot study or analysis) of data collected for this purpose (i.e., primary data) | 1.5(1,3.5) | 1(1,2.5) |
|  |  | Delphi study (to get consensus from experts on what population is most affected) | 4(4,5.25) | 2(2,5.375) |
|  |  | Jurisdictional scan (comparative analysis) to understand variability across populations in other jurisdictions | 5(1.75,8.25) | 3(3,3) |
|  |  | Cross-sectional study (survey, point-in-time or snapshot study or analysis) of data collected for other purposes (i.e., secondary data) | 5(2,8) | 4(4,4) |
|  |  | Social network analysis (mapping network analysis) to understand people’s interactions | 5.5(3,8.25) | 5(5,6.125) |
|  |  | Ecological study (population-based study, including spatial analysis) | 6.5(4,8) | 6(6,6.375) |
|  |  | Prospective cohort study of individual-level data (prospective longitudinal or panel study) | 8(6.75,8) | 7.5(5.5,8) |
|  |  | Qualitative deductive (from general to particular i.e., testing theory) methods to describe a phenomenon (e.g., qualitative description, narrative approaches) | 7(5.25,8) | 7(6.625,7) |
|  |  | Retrospective cohort study of individual-level data (retrospective or historical longitudinal, or panel study) | 8(7,8) | 9(7.875,9) |
|  |  | Case-control study (case-comparison study) | 12(12,12) | NA |
|  |  | Case reports (case series) | 12(12,12) | NA |
|  |  | Modelling to estimate an indicator (e.g., the impact of a problem on a given population) | 12(12,12) | NA |
|  | 3. Assessing the importance of a problem relative to other problems | Delphi study (to get consensus from experts) | 2.5(1.75,3.75) | 1(1,1.75) |
|  |  | Modelling to estimate an indicator that allows comparisons in common units (e.g., DALYs) | 2.5(1,4.75) | 2(2,2.75) |
|  |  | Ecological study (population-based study, including spatial analysis) | 4(3.5,5.75) | 3(3,4.5) |
|  |  | Multi-criteria (objective) decision analysis | 4.5(2,7) | 4(4,5.875) |
|  |  | Cross-sectional study (survey, point-in-time or snapshot study or analysis) of quantitative data (e.g., demographic information) | 6.5(4.75,7.125) | 5(5,5.75) |
|  |  | Case reports (case series) | 6.5(5,7.125) | 6.25(6,6.875) |
|  |  | Cross-sectional study (survey, point-in-time or snapshot study or analysis) of people's (affected by the problem) experiences (not asking about hypothetical scenarios) | 6.5(5.25,7.125) | 7(4,7) |
|  |  | Discrete choice experiment (stated preferences) | 6.5(5.5,7) | 7.5(6.625,8) |
|  |  | Qualitative deductive (from general to particular i.e., testing theory) methods to describe a phenomenon (e.g., qualitative description, narrative approaches) | 6.5(5.75,7) | 8.5(7.25,9) |
|  |  | Descriptive (non-qualitative) case study | 10(10,10) | NA |
| E. Understanding the causes and aggravating factors of a problem | 1. Identifying causes and/or aggravating factors of a problem | Prospective cohort study of individual-level data (prospective longitudinal or panel study) | 1(1,2) | 2(1,2) |
|  |  | Review to find causes or aggravating factors that have been identified by other studies (e.g., scoping review) | 3(1,4) | 3(1,3) |
|  |  | Retrospective cohort study of individual-level data (retrospective or historical longitudinal, or panel study) | 3(2,8.5) | 3(2,4) |
|  |  | Case-control study (case-comparison study) | 4(3,4) | 4(4,6.75) |
|  |  | Interrupted time-series analysis (including joint-point regression) | 8.5(8.5,9.5) | 6.5(5,7.5) |
|  |  | Regression discontinuity study (regression kink study or analysis) | 8.5(6,8.5) | 6.5(6,7.5) |
|  |  | Ecological study (population-based study, including spatial analysis) | 8.5(5,8.5) | 7(6.75,7) |
|  |  | Single before-and-after study of aggregated data (pre-post or pretest-posttest study) | 8.5(8.5,9.5) | 7(6.25,9) |
|  |  | Cross-sectional study (survey, point-in-time or snapshot study or analysis) of data collected for this purpose (i.e., primary data) | 8.5(6,8.5) | 7(6.75,8.5) |
|  |  | Instrumental variables study (two-stage least-squares study or regression) | 8.5(8.5,9.5) | 8(6.75,8) |
|  |  | Controlled before-and-after study of aggregated data (including difference-in-differences study and non-equivalent control group designs) | 13(13,13) | NA |
|  |  | Case reports (case series) | 13(13,13) | NA |
|  |  | Cross-sectional study (survey, point-in-time or snapshot study or analysis) of data collected for other purposes (i.e., secondary data) | 13(13,13) | NA |
|  | 2. Understanding the relative importance of causes and/or aggravating factors across population groups | Review to find causes or aggravating factors that have been identified by other studies (e.g., scoping review) | 4(3,9.5) | 2(1.25,2) |
|  |  | Prospective cohort study of individual-level data (prospective longitudinal or panel study) | 9(1,9.5) | 2.5(2,3.75) |
|  |  | Delphi studies (to get consensus from experts) | 9(3,9.5) | 3(3,5.25) |
|  |  | Case-control study (case-comparison study) | 3(3,9.5) | 5(1.75,6.375) |
|  |  | Cross-sectional study (survey, point-in-time or snapshot study or analysis) of data collected for this purpose (i.e., primary data) | 9(4,9.5) | 5.5(5,6) |
|  |  | Ecological study (population-based study, including spatial analysis) | 9(5,9.5) | 6(6,6) |
|  |  | Retrospective cohort study of individual-level data (retrospective or historical longitudinal, or panel study) | 9(2,9.5) | 6.75(3.875,7) |
|  |  | Regression discontinuity study (regression kink study or analysis) | 9.5(4,9.5) | 6.25(5.25,7.625) |
|  |  | Cross-sectional study (survey, point-in-time or snapshot study or analysis) of data collected for other purposes (i.e., secondary data) | 9.5(9,9.5) | NA |
|  |  | Multi-criteria (objective) decision analysis | 9.5(9,9.5) | NA |
|  |  | Case reports (case series) | 15(15,15) | NA |
|  |  | Controlled before-and-after study of aggregated data (including difference-in-differences study and non-equivalent control group designs) | 15(15,15) | NA |
|  |  | Instrumental variables study (two-stage least-squares study or regression) | 15(15,15) | NA |
|  |  | Interrupted time-series analysis (including joint-point regression) | 15(15,15) | NA |
|  |  | Single before-and-after study of aggregated data (pre-post or pretest-posttest study) | 15(15,15) | NA |
| F. Understanding the impacts of a problem | 1. Identifying impacts/spillover effects of a problem | Controlled before-and-after study of aggregated data (including difference-in-differences study and non-equivalent control group designs) | 3.5(1.5,7.75) | NA |
|  |  | Review to find causes or aggravating factors that have been identified by other studies (e.g., scoping review) | 3.5(3,7.75) | NA |
|  |  | Prospective cohort study of individual-level data (prospective longitudinal or panel study) | 5(1,9) | NA |
|  |  | Retrospective cohort study of individual-level data (retrospective or historical longitudinal, or panel study) | 6.5(2.5,9) | NA |
|  |  | Interrupted time-series analysis (including joint-point regression) | 7(2.75,9) | NA |
|  |  | Instrumental variables study (two-stage least-squares study or regression) | 7(3.5,9) | NA |
|  |  | Case-control study (case-comparison study) | 9(5.25,9.375) | NA |
|  |  | Modelling to predict fuure scenarios (e.g., system dynamics, ARIMA models, etc.) | 9(6.75,9) | NA |
|  |  | Cross-sectional study (survey, point-in-time or snapshot study or analysis) of data collected for this purpose (i.e., primary data) | 9(9,9.375) | NA |
|  |  | Single before-and-after study of aggregated data (pre-post or pretest-posttest study) | 9(9,9) | NA |
|  |  | Ecological study (population-based study, including spatial analysis) | 9(9,9) | NA |
|  |  | Regression discontinuity study (regression kink study or analysis) | 9(9,9) | NA |
|  |  | Case reports (case series) | 14(14,14) | NA |
|  |  | Cross-sectional study (survey, point-in-time or snapshot study or analysis) of data collected for other purposes (i.e., secondary data) | 14(14,14) | NA |
|  | 2. Prioritizing the most important impacts/spillover effects of a problem | Review to find causes or aggravating factors that have been identified by other studies (e.g., scoping review) | 4.5(3.25,6.5) | NA |
|  |  | Delphi studies (to get consensus from experts) | 5.5(1,10.375) | NA |
|  |  | Prospective cohort study of individual-level data (prospective longitudinal or panel study) | 7.25(2,9.875) | NA |
|  |  | Retrospective cohort study of individual-level data (retrospective or historical longitudinal, or panel study) | 7.75(3,9.875) | NA |
|  |  | Multi-criteria (objective) decision analysis | 9.75(5.375,10.375) | NA |
|  |  | Instrumental variables study (two-stage least-squares study or regression) | 9.75(5.375,10.375) | NA |
|  |  | Controlled before-and-after study of aggregated data (including difference-in-differences study and non-equivalent control group designs) | 9.75(4.625,10) | NA |
|  |  | Interrupted time-series analysis (including joint-point regression) | 9.75(5.375,10) | NA |
|  |  | Ecological study (population-based study, including spatial analysis) | 10(9.625,10.375) | NA |
|  |  | Modelling to predict future scenarios (e.g., system dynamics, ARIMA models, etc.) | 10(9.625,10.375) | NA |
|  |  | Regression discontinuity study (regression kink study or analysis) | 10(9.625,10.375) | NA |
|  |  | Case-control study (case-comparison study) | 10.25(9.625,10.5) | NA |
|  |  | Cross-sectional study (survey, point-in-time or snapshot study or analysis) of data collected for other purposes (i.e., secondary data) | 10.25(10,10.5) | NA |
|  |  | Cross-sectional study (survey, point-in-time or snapshot study or analysis) of data collected for this purpose (i.e., primary data) | 10.25(10,10.5) | NA |
|  |  | Case reports (case series) | 16(16,16) | NA |
|  |  | Single before-and-after study of aggregated data (pre-post or pretest-posttest study) | 16(16,16) | NA |

NA: Not available (questions were not included in the second round).

**S2 Table B. Ranking of study designs per Delphi round in stage II. Finding and selecting options to address a problem**

| **Goal** | **Type of question** | **Study design** | **Median rank (IQR) round 1** | **Median rank (IQR) round 2** |
| --- | --- | --- | --- | --- |
| A. Finding and understanding potential options. | 1. Scoping a list of potential options | Review to find options that have been used by other studies (e.g., scoping review) | 1(1,1) | NA |
|  |  | Jurisdictional scan (comparative analysis) to understand what options have been implemented by other jurisdictions | 2(2,2.25) | NA |
|  |  | Cross-sectional study (survey, point-in-time or snapshot study or analysis) of people’s opinions | 3.5(2.75,4.25) | NA |
|  |  | Ecological study (population-based study, including spatial analysis) | 3.75(3,4.625) | NA |
|  |  | Descriptive (non-qualitative) case study | 4.25(4,4.625) | NA |
|  | 2. Understanding the way potential options and their components work | Randomized-controlled study (randomized experiment or randomized trial) measuring intermediate outcomes | 2.5(2,4.875) | 1(1,3.5) |
|  |  | Review to identify existing frameworks (conceptual analysis) that explain how an intervention might work | 6.75(1.75,11.5) | 2(1,2) |
|  |  | Interrupted time-series analysis (including joint-point regression) measuring intermediate outcomes | 7.25(3.25,10.75) | 3(3,4.5) |
|  |  | Descriptive (non-qualitative) case study | 7.25(3.5,10.75) | 4(4,4) |
|  |  | Case reports (case series) | 11(8.125,11.5) | 5(5,6.75) |
|  |  | Qualitative deductive (from general to particular i.e., testing theory) methods to describe a phenomenon (e.g., qualitative description, narrative approaches) | 11(8.625,11.5) | 6(6,6.25) |
|  |  | Cross-sectional study (survey, point-in-time or snapshot study or analysis) of data collected for other purposes (i.e., secondary data) measuring intermediate outcomes | 11(8.875,11.5) | 7(7,7) |
|  |  | Prospective cohort study of individual-level data (prospective longitudinal or panel study) | 11(8.625,11.5) | 8(7,8) |
|  |  | Qualitative deductive (from general to particular i.e., testing theory) methods to describe/critically analyze a phenomenon (e.g., qualitative case studies) | 11.5(8.875,11.5) | 9(4.75,9) |
|  |  | Case-control study (case-comparison study) measuring intermediate outcomes | 19(19,19) | NA |
|  |  | Cross-sectional study (survey, point-in-time or snapshot study or analysis) of data collected for this purpose (i.e., primary data) measuring intermediate outcomes | 19(19,19) | NA |
|  |  | Controlled before-and-after study of aggregated data (including difference-in-differences study and non-equivalent control group designs) measuring intermediate outcomes | 19(19,19) | NA |
|  |  | Ecological study (population-based study, including spatial analysis) | 19(19,19) | NA |
|  |  | Instrumental variables study (two-stage least-squares study or regression) measuring intermediate outcomes | 19(19,19) | NA |
|  |  | Modelling to predict the mechanism of action of a given intervention | 19(19,19) | NA |
|  |  | Retrospective cohort study of individual-level data (retrospective or historical longitudinal, or panel study) | 19(19,19) | NA |
|  |  | Regression discontinuity study (regression kink study or analysis) | 19(19,19) | NA |
|  |  | Single before-and-after study of aggregated data (pre-post or pretest-posttest study) measuring intermediate outcomes | 19(19,19) | NA |
|  |  | Social network analysis (mapping network analysis) | 19(19,19) | NA |
| B. Assessing the expected impact of options | 1. Assessing the feasibility of an option | Delphi studies (to get consensus from experts) | 3.5(3,6.25) | 1(1,2) |
|  |  | Jurisdictional scan (comparative analysis) to understand the feasibility of the option elsewhere | 5.25(1.25,7.5) | 2(1,2) |
|  |  | Discrete choice experiment (stated preferences) | 6.5(3.25,7.5) | 3(3,5.75) |
|  |  | Modelling to predict whether the option will be feasible (e.g., system dynamics, ARIMA models, etc.) | 7.25(3.25,7.5) | 4(4,6.75) |
|  |  | Qualitative deductive (from general to particular i.e., testing theory) methods to describe/critically analyze a phenomenon (e.g., qualitative case studies) | 7.25(2.5,7.5) | 5(5,6.75) |
|  |  | Cross-sectional study (survey, point-in-time or snapshot study or analysis) of people’s opinions (not asking about hypothetical scenarios) | 7.25(2,7.5) | 6(6,6.25) |
|  |  | Descriptive (non-qualitative) pilot case study | 7.5(3.5,7.5) | 7(6.5,7) |
|  |  | Qualitative deductive (from general to particular i.e., testing theory) methods to describe a phenomenon (e.g., qualitative description, narrative approaches) | 7.5(4.75,7.5) | 7(6.5,8) |
|  |  | Social network analysis (mapping network analysis) | 7.5(4.75,7.5) | 9(6.75,9) |
|  |  | Randomized-controlled study (randomized experiment or randomized trial), including pilot RCTs | 7.5(7.125,7.5) | NA |
|  | 2. Assessing the benefits and early-and-frequently occurring harms of an option    3. Assessing late-occurring harms and risks of an option | Randomized-controlled study (randomized experiment or randomized trial) | 1(1,7.25) | 1(1,1) |
|  |  | Controlled before-and-after study of aggregated data (including difference-in-differences study and non-equivalent control group designs) | 3(2.25,8.125) | 2(2,2) |
|  |  | Interrupted time-series analysis (including joint-point regression) | 7.5(4,9.5) | 3(3,3) |
|  |  | Retrospective cohort study of individual-level data (retrospective or historical longitudinal, or panel study) | 8(4.25,9.5) | 4(4,5.5) |
|  |  | Instrumental variables study (two-stage least-squares study or regression) | 8(4.25,9.5) | 5(5,5.75) |
|  |  | Prospective cohort study of individual-level data (prospective longitudinal or panel study) | 8.5(3.75,9.5) | 6(5.25,6) |
|  |  | Regression discontinuity study (regression kink study or analysis) | 9.25(3.5,9.5) | 7(7,7) |
|  |  | Modelling to predict or estimate the benefits of an intervention (e.g., system dynamics, ARIMA models, etc.) | 9.5(6.125,9.875) | 8(8,8) |
|  |  | Case-control study (case-comparison study) | 9.25(9,9.5) | NA |
|  |  | Ecological study (population-based study, including spatial analysis) | 9.5(9.125,9.875) | NA |
|  |  | Case reports (case series) | 9.5(9.5,10) | NA |
|  |  | Cross-sectional study (survey, point-in-time or snapshot study or analysis) of data collected for this purpose (i.e., primary data) | 9.5(9.125,9.875) | NA |
|  |  | Single before-and-after study of aggregated data (pre-post or pretest-posttest study) | 9.5(9.5,10) | NA |
|  |  | Cross-sectional study (survey, point-in-time or snapshot study or analysis) of data collected for other purposes (i.e., secondary data) | 9.5(9.5,10) | NA |
|  |  | Retrospective cohort study of individual-level data (retrospective or historical longitudinal, or panel study), including databases of adverse event reporting (e.g., pharmacovigilance) | 4(3,9.375) | 1(1,3.5) |
|  |  | Randomized-controlled study (randomized experiment or randomized trial) | 5.5(1,9.5) | 2(1,2) |
|  |  | Prospective cohort study of individual-level data (prospective longitudinal or panel study) | 6.5(2,9.5) | 2(2,3) |
|  |  | Case-control study (case-comparison study) | 8(1.75,9.5) | 4(4,5.5) |
|  |  | Instrumental variables study (two-stage least-squares study or regression) | 9.25(4,9.5) | 5(4,5) |
|  |  | Controlled before-and-after study of aggregated data (including difference-in-differences study and non-equivalent control group designs) | 9.5(5.25,9.5) | 6(6,7) |
|  |  | Ecological study (population-based study, including spatial analysis) | 9.5(4.5,9.5) | 7(7,7) |
|  |  | Interrupted time-series analysis (including joint-point regression) | 9.5(6.75,9.5) | 8(7,8) |
|  |  | Case reports (case series) | 9.5(8.25,9.5) | 9(8,9) |
|  |  | Modelling to predict or estimate the harms and risks of an intervention (e.g., system dynamics, ARIMA models, etc.) | 9.5(9.125,9.5) | NA |
|  |  | Regression discontinuity study (regression kink study or analysis) | 9.5(9.125,9.5) | NA |
|  |  | Cross-sectional study (survey, point-in-time or snapshot study or analysis) of data collected for other purposes (i.e., secondary data) | 9.5(9.125,9.5) | NA |
|  |  | Cross-sectional study (survey, point-in-time or snapshot study or analysis) of data collected for this purpose (i.e., primary data) | 9.5(9.5,9.5) | NA |
|  |  | Single before-and-after study of aggregated data (pre-post or pretest-posttest study) | 9.5(9.5,9.5) | NA |
|  | 4. Assessing the costs and resource use of an option | Modelling to estimate the cost of an option | 2(2,3.75) | 1(1,1) |
|  |  | Jurisdictional scan (comparative analysis) to understand the costs in other jurisdictions | 9.75(3.25,10.5) | 2(2,3) |
|  |  | Case reports (case series) | 6.75(3,10.5) | 3(2,7) |
|  |  | Cross-sectional study (survey, point-in-time or snapshot study or analysis) of data collected for this purpose (i.e., primary data) | 9.75(5.25,10.5) | 4(4,6) |
|  |  | Delphi studies (to get consensus from experts) | 10(4.5,10.5) | 5(5,7.25) |
|  |  | Cross-sectional study (survey, point-in-time or snapshot study or analysis) of data collected for other purposes (i.e., secondary data) | 10.25(5.375,10.5) | 6(6,7.25) |
|  |  | Prospective cohort study of individual-level data (prospective longitudinal or panel study) | 10.25(7.625,10.5) | 7(5.5,7.75) |
|  |  | Instrumental variables study (two-stage least-squares study or regression) | 10.25(9.625,10.5) | 7.5(7,8.75) |
|  |  | Retrospective cohort study of individual-level data (retrospective or historical longitudinal, or panel study), including databases of adverse event reporting (e.g., pharmacovigilance) | 10.5(9.625,10.5) | 8.5(7.25,9.75) |
|  |  | Controlled before-and-after study of aggregated data (including difference-in-differences study and non-equivalent control group designs) | 10.5(9.625,10.5) | 9(8,9.25) |
|  |  | Randomized-controlled study (randomized experiment or randomized trial) | 10.5(9.625,10.5) | 9.5(8,11) |
|  |  | Case-control study (case-comparison study) | 10.5(10.125,10.5) | NA |
|  |  | Ecological study (population-based study, including spatial analysis) | 10.5(10.125,10.5) | NA |
|  |  | Regression discontinuity study (regression kink study or analysis) | 10.5(10.125,10.5) | NA |
|  |  | Single before-and-after study of aggregated data (pre-post or pretest-posttest study) | 10.5(10.5,10.5) | NA |
|  |  | Interrupted time-series analysis (including joint-point regression) | 10.5(10.5,10.5) | NA |
|  | 5. Assessing the efficiency in the use of resources | Economic evaluations (cost-effectiveness, cost-utility, cost-benefit analyses) | 1(1,1) | NA |
|  |  | Jurisdictional scan (comparative analysis) to understand whether the option was efficient in other jurisdictions | 2(2,2.5) | NA |
|  |  | Delphi studies (to get consensus from experts) | 3(2.5,3) | NA |
|  | 6. Identifying equity, ethical, social and human rights impacts of an option | Delphi studies (to get consensus from experts) | 2(2,3.75) | 1(1,1.5) |
|  |  | Cross-sectional study (survey, point-in-time or snapshot study or analysis) of people’s experiences (not asking about hypothetical scenarios) | 2.5(1,3.75) | 2(2,2) |
|  |  | Qualitative deductive (from general to particular i.e., testing theory) methods to describe/critically analyze a phenomenon (e.g., qualitative case studies) | 3.5(2,5.875) | 3(2.5,3) |
|  |  | Qualitative deductive (from general to particular i.e., testing theory) methods to describe a phenomenon (e.g., qualitative description, narrative approaches) | 4(3,5.875) | 4(4,4) |
|  |  | Descriptive (non-qualitative) case study | 5.25(3,5.5) | 5(5,5) |
|  |  | Qualitative inductive (from particular to general i.e., creating theory) methods to interpret/critically analyze a phenomenon (e.g., ethnographic approaches, phenomenology) | 5.5(3.25,6) | 6(5.75,6) |
|  |  | Discrete choice experiment (stated preferences) | 6(5.625,6) | 7(6.25,7) |
|  | 7. Assessing the acceptability of an option | Discrete choice experiment (stated preferences) | 2.5(1.25,4) | 1(1,1.5) |
|  |  | Qualitative deductive (from general to particular i.e., testing theory) methods to describe/critically analyze a phenomenon (e.g., qualitative case studies) | 2.5(2,4) | 2(2,2) |
|  |  | Qualitative deductive (from general to particular i.e., testing theory) methods to describe a phenomenon (e.g., qualitative description, narrative approaches) | 3(2,4) | 3(3,3) |
|  |  | Cross-sectional study (survey, point-in-time or snapshot study or analysis) of people’s experiences (not asking about hypothetical scenarios) | 3(2,3.75) | 4(4,4) |
|  |  | Randomized-controlled study (randomized experiment or randomized trial) measuring people’s acceptability | 4.75(4,5) | NA |
| C. Maximizing the expected impact of options | 1. Adjusting options and enabling factors to maximize impact | Randomized-controlled study (randomized experiment or randomized trial) to compare different forms of the same intervention | 1(1,4.25) | 1(1,1) |
|  |  | Controlled before-and-after study of aggregated data (including difference-in-differences study and non-equivalent control group designs) | 4(2,9.5) | 2(2,4.25) |
|  |  | Interrupted time-series analysis (including joint-point regression) | 6.75(4,9.5) | 4(4,5.5) |
|  |  | Instrumental variables study (two-stage least-squares study or regression) | 5.5(3.75,9.5) | 4(3,6.5) |
|  |  | Randomized-controlled study (randomized experiment or randomized trial) using posthoc comparisons | 9.5(2,9.875) | 6(3,6) |
|  |  | Prospective cohort study of individual-level data (prospective longitudinal or panel study) | 9.5(7,9.5) | 6(4,8) |
|  |  | Modelling to predict or estimate the impact of an intervention (e.g., system dynamics, ARIMA models, etc.) | 6.75(3,10.25) | 6.25(5,7.875) |
|  |  | Regression discontinuity study (regression kink study or analysis) | 9.5(3,9.625) | 7(7,7.375) |
|  |  | Retrospective cohort study of individual-level data (retrospective or historical longitudinal, or panel study) | 9.5(7.5,9.5) | 8.5(7.625,9) |
|  |  | Case-control study (case-comparison study) | 9.5(8.375,9.875) | 9.5(8.25,10) |
|  |  | Cross-sectional study (survey, point-in-time or snapshot study or analysis) of data collected for this purpose (i.e., primary data) | 9.5(9,9.5) | NA |
|  |  | Cross-sectional study (survey, point-in-time or snapshot study or analysis) of data collected for other purposes (i.e., secondary data) | 9.5(9.125,9.875) | NA |
|  |  | Single before-and-after study of aggregated data (pre-post or pretest-posttest study) | 9.5(9.5,9.625) | NA |
|  |  | Case reports (case series) | 9.5(9.5,9.875) | NA |
|  | 2. Finding population groups, settings and contexts to focusing options | Case-control study (case-comparison study) | 4(2.5,9.5) | 1.5(1,2.75) |
|  |  | Prospective cohort study of individual-level data (prospective longitudinal or panel study) | 4(2.5,7.25) | 2(1.25,2) |
|  |  | Controlled before-and-after study of aggregated data (including difference-in-differences study and non-equivalent control group designs) | 7(3.5,9.5) | 3(3,5.25) |
|  |  | Randomized-controlled study (randomized experiment or randomized trial) using subgroup comparisons. | 9.5(1,9.5) | 4(2.5,4) |
|  |  | Case reports (case series) | 9.5(4.5,9.5) | 5(5,6.875) |
|  |  | Instrumental variables study (two-stage least-squares study or regression) | 9.5(6.75,9.5) | 6(6,7.125) |
|  |  | Ecological study (population-based study, including spatial analysis) | 9.5(5,9.5) | 7(7,7.375) |
|  |  | Cross-sectional study (survey, point-in-time or snapshot study or analysis) of data collected for other purposes (i.e., secondary data) | 9.5(2.5,9.75) | 7.75(7.125,8) |
|  |  | Cross-sectional study (survey, point-in-time or snapshot study or analysis) of data collected for this purpose (i.e., primary data) | 9.5(5.75,10.25) | 8.5(7.625,9) |
|  |  | Retrospective cohort study of individual-level data (retrospective or historical longitudinal, or panel study) | 9.5(7,9.5) | 8.75(7.125,10) |
|  |  | Modelling to predict or estimate the impact of an intervention (e.g., system dynamics, ARIMA models, etc.) | 9.5(7,9.5) | 11(10.25,11) |
|  |  | Interrupted time-series analysis (including joint-point regression) | 9.5(9.5,9.5) | NA |
|  |  | Regression discontinuity study (regression kink study or analysis) | 9.5(9.5,10.25) | NA |
|  |  | Single before-and-after study of aggregated data (pre-post or pretest-posttest study) | 9.5(9.5,9.5) | NA |
| D. Contributing to prioritize and select options | 1. Creating packages of options | Evidence synthesis of studies evaluating the impact of single interventions to analyze the combined effect of packages. | 2(1,3.5) | 1(1,1) |
|  |  | Randomized-controlled study (randomized experiment or randomized trial) to compare packages of interventions in different arms | 3(1.5,7.5) | 2(2,2) |
|  |  | Randomized-controlled study (randomized experiment or randomized trial) using posthoc comparisons. | 6(4,11.5) | 3(3,3) |
|  |  | Delphi study (to get consensus from experts) | 7(3.5,12) | 4(4,4) |
|  |  | Modelling to predict or estimate the impact of interventions and packages of interventions (e.g., system dynamics, ARIMA models, etc.) | 11(4,12) | 5(5,5) |
|  |  | Controlled before-and-after study of aggregated data (including difference-in-differences study and non-equivalent control group designs) | 12(7,12) | 6(6,6) |
|  |  | Economic evaluations (cost-effectiveness, cost-utility, cost-benefit analyses) | 12(7,12) | 7(7,8.25) |
|  |  | Retrospective cohort study of individual-level data (retrospective or historical longitudinal, or panel study) | 12(11.5,12) | 8(8,8.75) |
|  |  | Ecological study (population-based study, including spatial analysis) | 12(11.5,12) | 9(9,9.25) |
|  |  | Case-control study (case-comparison study) | 12(11.5,12) | 10(9.75,10) |
|  |  | Interrupted time-series analysis (including joint-point regression) | 12(10.5,12) | 11(10.5,11) |
|  |  | Prospective cohort study of individual-level data (prospective longitudinal or panel study) | 12(11,12) | 12(11,12) |
|  |  | Discrete choice experiment (stated preferences) | 12(11.5,13) | 13(11.5,13) |
|  |  | Multi-criteria (objective) decision analysis | 12(11.5,13) | 14(12,14) |
|  |  | Cross-sectional study (survey, point-in-time or snapshot study or analysis) | 12(12,12) | NA |
|  |  | Instrumental variables study (two-stage least-squares study or regression) | 12(12,12.5) | NA |
|  |  | Case reports (case series) | 12(12,13) | NA |
|  |  | Regression discontinuity study (regression kink study or analysis) | 12(12,13) | NA |
|  |  | Single before-and-after study of aggregated data (pre-post or pretest-posttest study) | 12(12,13) | NA |
|  | 2. Creating a ranking of options | Economic evaluations (cost-effectiveness, cost-utility, cost-benefit analyses) to create a ranked list of options | 1(1,2) | NA |
|  |  | Ranking type Delphi study (to get consensus from experts) | 2(2,2.5) | NA |
|  |  | Multi-criteria (objective) decision analysis to create a ranked list of options | 2(2,3) | NA |
|  |  | Discrete choice experiment (stated preferences) | 4(4,4.75) | NA |
|  |  | Jurisdictional scan (comparative analysis) to understand what other jurisdictions have ranked. | 4.5(4,5.25) | NA |
|  |  | Qualitative deductive (from general to particular i.e., testing theory) methods to describe a phenomenon (e.g., qualitative description, narrative approaches) | 5.5(5.25,5.75) | NA |

NA: Not available (questions were not included in the second round).

**S2 Table C. Ranking of study designs per Delphi round in stage III. Implementing or scaling-up an option**

| **Goal** | **Type of question** | **Study design** | **Median rank (IQR) round 1** | **Median rank (IQR) round 2** |
| --- | --- | --- | --- | --- |
| A. Setting up a sustainable implementation process by identifying barriers, facilitators and implementation strategies | 1. Identifying and understanding barriers and implementation strategies to deal with them | Review to find barriers and implementation strategies that have been used by other studies (e.g., scoping review) | 1.5(1,2.75) | 1(1,1) |
|  |  | Cross-sectional study (survey, point-in-time or snapshot study or analysis) of people's experiences | 3(3,3) | 2(2,2.5) |
|  |  | Qualitative deductive (from general to particular i.e., testing theory) methods to describe/critically analyze a phenomenon (e.g., qualitative case studies) | 6.5(3,7) | 4(3.5,4.75) |
|  |  | Social network analysis (mapping network analysis) to identify barriers and implementation strategies | 4(4,6.25) | 5(3,5.75) |
|  |  | Qualitative deductive (from general to particular i.e., testing theory) methods to describe a phenomenon (e.g., qualitative description, narrative approaches) | 7(3.25,7) | 5(5,5.25) |
|  |  | Jurisdictional scan (comparative analysis) to understand what barriers and implementation | 7(4,7) | 6(3,6) |
|  |  | Qualitative inductive (from particular to general i.e., creating theory) methods to describe a phenomenon (e.g., grounded theory) | 7(5.5,7) | 7(5,7) |
|  |  | Modelling to predict the mechanism of action of a given barrier or implementation strategy | 7(7,7) | NA |
|  |  | Descriptive (non-qualitative) case study | 7(7,7) | NA |
|  | 2. Identifying and understanding facilitators and implementation strategies to take advantage of them | Review to find facilitators and implementation strategies that have been used by other studies (e.g., scoping review) | 1.5(1,2.75) | 1(1,2.5) |
|  |  | Cross-sectional study (survey, point-in-time or snapshot study or analysis) of people's experiences | 3(2.25,3.75) | 2(2,2.5) |
|  |  | Qualitative deductive (from general to particular i.e., testing theory) methods to describe/critically analyze a phenomenon (e.g., qualitative case studies) | 5.5(3.25,7) | 3(3,4.25) |
|  |  | Qualitative deductive (from general to particular i.e., testing theory) methods to describe a phenomenon (e.g., qualitative description, narrative approaches) | 6.5(3,7) | 5(3.5,5) |
|  |  | Social network analysis (mapping network analysis) to identify facilitators and implementation strategies | 5.5(4,7) | 5(4,5.75) |
|  |  | Jurisdictional scan (comparative analysis) to understand what facilitators and implementation strategies have been identified by other jurisdictions | 7(4,7) | 6(3.5,6) |
|  |  | Qualitative inductive (from particular to general i.e., creating theory) methods to describe a phenomenon (e.g., grounded theory) | 7(5.5,7) | 7(5.75,7) |
|  |  | Descriptive (non-qualitative) case study | 7(7,7) | NA |
|  |  | Modelling to predict the mechanism of action of a given facilitator or implementation strategy | 7(7,7) | NA |
|  | 3. Prioritizing barriers, facilitators and implementation strategies | Delphi study (to get consensus from experts) | 1.5(1,3.5) | 1(1,1.5) |
|  |  | Discrete choice experiment (stated preferences) | 3(2,8.875) | 2(2,6.5) |
|  |  | Multi-criteria (objective) decision analysis | 3.5(3,8.875) | 3(3,8) |
|  |  | Cross-sectional study (survey, point-in-time or snapshot study or analysis) | 8.25(2.75,11.5) | 4(4,7) |
|  |  | Evidence synthesis of studies evaluating the impact of single interventions to analyze the combined effect of packages. | 11(4.875,11.5) | 6(3.5,7) |
|  |  | Retrospective cohort study of individual-level data (retrospective or historical longitudinal, or panel study) | 10.25(4.75,11.25) | 7(5,8) |
|  |  | Randomized-controlled study (randomized experiment or randomized trial) of implementation issues (e.g., implementation trial) | 11(6.375,11.5) | 7(7,8) |
|  |  | Single before-and-after study of aggregated data (pre-post or pretest-posttest study) | 11.5(10.75,11.5) | 8(8,8.5) |
|  |  | Modelling to predict or estimate the importance of barriers, facilitators and implementation strategies | 11.5(10.75,11.5) | 9(8.5,9) |
|  |  | Economic evaluations (cost-effectiveness, cost-utility, cost-benefit analyses) | 11.5(10.75,11.875) | 9(8,13) |
|  |  | Controlled before-and-after study of aggregated data (including difference-in-differences study and non-equivalent control group designs) | 11.5(10.75,11.5) | 9(8.5,12) |
|  |  | Prospective cohort study of individual-level data (prospective longitudinal or panel study) | 11.5(10.75,11.5) | 10(6,10) |
|  |  | Case-control study (case-comparison study) | 11.5(10.75,11.875) | 10(8.5,11) |
|  |  | Ecological study (population-based study, including spatial analysis) | 11.5(11.125,11.5) | NA |
|  |  | Interrupted time-series analysis (including joint-point regression) | 11.5(11.5,11.875) | NA |
|  |  | Regression discontinuity study (regression kink study or analysis) | 11.5(11.5,11.875) | NA |
|  |  | Instrumental variables study (two-stage least-squares study or regression) | 11.5(11.5,11.875) | NA |
|  |  | Case reports (case series) | 11.5(11.5,11.875) | NA |
| B. Planning and describing the implementation of an option | 1. Identifying who has to do what to implement an option | Delphi studies (to get consensus from experts) | 2(2,2) | 1(1,1) |
|  |  | Cross-sectional study (survey, point-in-time or snapshot study or analysis) of people's experiences | 3(1,6) | 2(2,2) |
|  |  | Qualitative inductive (from particular to general i.e., creating theory) methods to describe a phenomenon (e.g., grounded theory) | 6(5,7) | 3(3,4.75) |
|  |  | Qualitative deductive (from general to particular i.e., testing theory) methods to describe a phenomenon (e.g., qualitative description, narrative approaches) | 6(4,6) | 4(4,4) |
|  |  | Qualitative deductive (from general to particular i.e., testing theory) methods to describe/critically analyze a phenomenon (e.g., qualitative case studies) | 6(4,7) | 5(5,5.5) |
|  |  | Social network analysis (mapping network analysis) | 6(3,7) | 6(6,6) |
|  |  | Descriptive (non-qualitative) case study | 6(4,7) | 7(6.75,7) |
|  |  | Jurisdictional scan (comparative analysis) to understand what other jurisdictions have identified as behavioural variables | 7(6,7) | 8(8,8) |
|  |  | Modelling to predict the mechanism of action of a given barrier or implementation strategy | 7(6,7) | 9(9,9) |
|  | 2. Identifying the context in which the option could be implemented | Jurisdictional scan (comparative analysis) to understand what other jurisdictions have identified as contextual variables | 2(1,3.5) | 1(1,1) |
|  |  | Qualitative deductive (from general to particular i.e., testing theory) methods to describe a phenomenon (e.g., qualitative description, narrative approaches) | 3(1.75,4) | 2(2,3.5) |
|  |  | Cross-sectional study (survey, point-in-time or snapshot study or analysis) of people's experiences | 4(2,6) | 3(3,3.5) |
|  |  | Delphi studies (to get consensus from experts) | 4(2.5,5.25) | 4(3,4) |
|  |  | Qualitative inductive (from particular to general i.e., creating theory) methods to describe a phenomenon (e.g., grounded theory) | 4.5(3.75,5.25) | 5(5,5) |
|  |  | Qualitative deductive (from general to particular i.e., testing theory) methods to describe/critically analyze a phenomenon (e.g., qualitative case studies) | 5(4.25,5.25) | 6(6,6) |
|  |  | Descriptive (non-qualitative) pilot case study | 6(5.75,6.25) | 7(7,7) |
|  | 3. Describing whether implementation of an option is underway and at what stage level | Cross-sectional study (survey, point-in-time or snapshot study or analysis) of people's experiences | 2(1,3.625) | 1(1,1) |
|  |  | Descriptive (not predicting) time-series analysis (including trend analysis) | 2(2,2) | 2(2,3.75) |
|  |  | Qualitative deductive (from general to particular i.e., testing theory) methods to describe/critically analyze a phenomenon (e.g., qualitative case studies) | 3.75(3,4.625) | 3(2.5,3) |
|  |  | Qualitative deductive (from general to particular i.e., testing theory) methods to describe a phenomenon (e.g., qualitative description, narrative approaches, documentary review of public speeches, etc.) | 4.25(4,4.625) | 4(4,4.75) |
|  |  | Delphi studies (to get consensus from experts) | 4.75(3.625,5) | 5(4.75,5) |
|  |  | Descriptive (non-qualitative) pilot case study | 5(3.625,5.625) | 6(4.75,6) |

NA: Not available (questions were not included in the second round).

**S2 Table D. Ranking of study designs per Delphi round in stage IV. Monitoring implementation and evaluating impacts**

| **Goal** | **Type of question** | **Study design** | **Median rank (IQR) round 1** | **Median rank (IQR) round 2** |
| --- | --- | --- | --- | --- |
| A. Identifying measurement strategies for populations and outcomes | 1. Identifying instruments to ascertain populations | Review to find measurement strategies that have been used by other studies (e.g., scoping review) | 3(1,5) | 1(1,3) |
|  |  | Cross-sectional study (survey, point-in-time or snapshot study or analysis) of people’s opinions on measurement strategies | 2(1,3) | 2(1,3) |
|  |  | Jurisdictional scan (comparative analysis) to understand what measurement strategies have been used by other jurisdictions | 2(2,2.5) | 2(2,2) |
|  |  | Descriptive (non-qualitative) case study | 4(3.75,4) | 4(4,5) |
|  |  | Qualitative deductive (from general to particular i.e., testing theory) methods to describe a phenomenon (e.g., qualitative description, narrative approaches) | 4(2.75,5) | 5(4,5) |
|  | 2. Choosing the most accurate instruments to ascertain populations | Delphi study (to get consensus from experts) | 2(2,4) | 1(1,1) |
|  |  | Multi-criteria (objective) decision analysis | 3(2,3) | 2(2,2) |
|  |  | Modelling to compare different measurement strategies | 5(4,5) | 3(3,3) |
|  |  | Cross-sectional study (survey, point-in-time or snapshot study or analysis) of data collected for this purpose (i.e., primary data) | 6.5(3,7.5) | 4(4,4) |
|  |  | Cross-sectional study (survey, point-in-time or snapshot study or analysis) of data collected for other purposes (i.e., secondary data) | 6.5(4,8) | 5(5,5) |
|  |  | Case-control study (case-comparison study) | 7(6.5,7.5) | 6(6,6) |
|  |  | Randomized-controlled study (randomized experiment or randomized trial) | 7.5(6.5,8) | 7(7,7) |
|  |  | Case reports (case series) | 7.5(6.5,8) | 8(8,8.5) |
|  |  | Retrospective cohort study of individual-level data (retrospective or historical longitudinal, or panel study) | 7.5(6.5,8) | 9(8.5,9) |
|  |  | Prospective cohort study of individual-level data (prospective longitudinal or panel study) | 7.5(6.5,8) | 10(8.5,10) |
|  | 3. Identifying measurement instruments for outcomes of interest | Review to find measurement strategies that have been used by other studies (e.g., scoping review) | 1.5(1,2.5) | 1(1,1) |
|  |  | Jurisdictional scan (comparative analysis) to understand what measurement strategies have been used by other jurisdictions | 2(1.75,2.25) | 2(2,2) |
|  |  | Descriptive (non-qualitative) case study | 3.5(2.75,4) | 3(3,3.75) |
|  |  | Cross-sectional study (survey, point-in-time or snapshot study or analysis) of people’s opinions on measurement strategies | 4(2.5,5) | 4(4,4) |
|  |  | Qualitative deductive (from general to particular i.e., testing theory) methods to describe a phenomenon (e.g., qualitative description, narrative approaches) | 4.5(3.75,5) | 5(4.25,5) |
|  | 4. Determining the best instruments to measure outcomes of interest | Delphi study (to get consensus from experts) | 2(1,4) | 1(1,3.25) |
|  |  | Multi-criteria (objective) decision analysis | 3(2,7.5) | 2(2,4.25) |
|  |  | Cross-sectional study (survey, point-in-time or snapshot study or analysis) of data collected for this purpose (i.e., primary data) | 4(2,4) | 3(2.25,3) |
|  |  | Cross-sectional study (survey, point-in-time or snapshot study or analysis) of data collected for other purposes (i.e., secondary data) | 5(2,7.5) | 4(3.25,4) |
|  |  | Modelling to compare different measurement strategies | 7.5(3,8) | 5.25(5,5.875) |
|  |  | Case reports (case series) | 7.5(5,7.5) | 6(5.625,6) |
|  |  | Prospective cohort study of individual-level data (prospective longitudinal or panel study) | 7.5(6,7.5) | 7(5.875,7) |
|  |  | Nominal groups technique (NGT) | NA | 8(6.5,8) |
|  |  | Case-control study (case-comparison study) | 7.5(7,7.5) | NA |
|  |  | Retrospective cohort study of individual-level data (retrospective or historical longitudinal, or panel study) | 7.5(7.5,7.5) | NA |
|  |  | Randomized-controlled study (randomized experiment or randomized trial) | 7.5(7.5,8) | NA |
| B. Monitoring and evaluating populations and outcomes of interests | 1. Measuring the impact of an option or implementation strategy | Randomized-controlled study (randomized experiment or randomized trial), including pragmatic trials | 1(1,1) | NA |
|  |  | Controlled before-and-after study of aggregated data (including difference-in-differences study and non-equivalent control group designs) | 3.5(2.25,4) | NA |
|  |  | Interrupted time-series analysis (including joint-point regression) | 3.5(3,4) | NA |
|  |  | Regression discontinuity study (regression kink study or analysis) | 5(2.5,8.625) | NA |
|  |  | Instrumental variables study (two-stage least-squares study or regression) | 7.25(5,9.5) | NA |
|  |  | Modelling the impact of an intervention on outcomes that are not observable. | 7.75(5.25,9.5) | NA |
|  |  | Case-control study (case-comparison study) | 9.5(8.375,9.5) | NA |
|  |  | Ecological study (population-based study, including spatial analysis) | 9.5(9.5,10.25) | NA |
|  |  | Retrospective cohort study of individual-level data (retrospective or historical longitudinal, or panel study) | 9.5(9.5,10.25) | NA |
|  |  | Case reports (case series) | 9.5(9.5,10.25) | NA |
|  |  | Prospective cohort study of individual-level data (prospective longitudinal or panel study) | 9.75(9.5,10.375) | NA |
|  |  | Cross-sectional study (survey, point-in-time or snapshot study or analysis) of data collected for other purposes (i.e., secondary data) | 10(9.5,10.5) | NA |
|  |  | Single before-and-after study of aggregated data (pre-post or pretest-posttest study) | 10(9.5,10.5) | NA |
|  |  | Cross-sectional study (survey, point-in-time or snapshot study or analysis) of data collected for this purpose (i.e., primary data) | 10(9.5,10.5) | NA |
|  | 2. Interpreting the findings of measuring the impact of an option or implementation strategy | Cross-sectional study (survey, point-in-time or snapshot study or analysis) of people's experiences (not asking about hypothetical scenarios) | 2(1,2) | 1(1,1) |
|  |  | Qualitative deductive (from general to particular i.e., testing theory) methods to describe a phenomenon (e.g., qualitative description, narrative approaches) | 3(2,3) | 2(2,2) |
|  |  | Descriptive (non-qualitative) case study | 3(2,5) | 3(3,3) |
|  |  | Qualitative deductive (from general to particular i.e., testing theory) methods to describe/critically analyze a phenomenon (e.g., qualitative case studies) | 4(3,4) | 4(4,4) |
|  |  | Delphi study (to get consensus from experts) | 5(3,5.5) | 5(5,5) |
|  |  | Discrete choice experiment (stated preferences) | 5(5,5.5) | NA |

NA: Not available (questions were not included in the second round).
